# Outcomes of Newly Diagnosed Acute Myeloid Leukemia Patients Treated With Hypomethylating Agents With or Without Venetoclax: A Propensity Score-Adjusted Cohort Study

**DOI:** 10.3389/fonc.2022.858202

**Published:** 2022-03-31

**Authors:** Moaath K. Mustafa Ali, Elizabeth M. Corley, Hanan Alharthy, Kathryn A. F. Kline, Jennie Y. Law, Seung Tae Lee, Sandrine Niyongere, Vu H. Duong, Ashkan Emadi, Maria R. Baer

**Affiliations:** ^1^ Greenebaum Comprehensive Cancer Center, University of Maryland, Baltimore, MD, United States; ^2^ Department of Medicine, University of Maryland School of Medicine, Baltimore, MD, United States; ^3^ School of Medicine, University of Maryland, Baltimore, MD, United States; ^4^ Department of Pharmacology, University of Maryland School of Medicine, Baltimore, MD, United States; ^5^ Translational Genomics Laboratory, Greenebaum Comprehensive Cancer Center, University of Maryland, Baltimore, MD, United States

**Keywords:** acute myeloid leukemia, azacitidine, decitabine (451668), venetoclax (ABT-199), outcomes, overall survival (OS), event-free survival (EFS)

## Abstract

There is a deficiency of real-world data on the impact of combining venetoclax (VEN) with hypomethylating agents (HMAs) in newly diagnosed acute myeloid leukemia (AML) patients. We conducted a single-center, propensity-adjusted retrospective cohort study to compare composite complete remission (CCR) rates, median overall survival (m-OS) and median event-free survival (m-EFS). A total of 170 adult AML patients were treated with first-line azacitidine (AZA) or decitabine (DEC) +/- VEN. Median age was 71 years and 99 (58%) were male. Median follow-up in HMA and HMA-VEN groups was 79 and 21 months. Treatments included AZA alone (n=35, 21%), DEC alone (n=84, 49%), AZA-VEN (n=24, 14%) and DEC-VEN (n=27, 16%). VEN improved CCR rates to HMAs overall (52% vs. 27%, P<0.05) and to AZA (54% vs. 10%, P<0.05), but not to DEC (43% vs. 32%, P=0.35); it did not improve OS, and only improved EFS for AZA (10.5 vs. 3.8 months, P<0.05). CCR rates were lower with AZA than with DEC (13% vs. 33%, P<0.05), but OS and EFS were not different statistically. CCR rates did not differ for AZA-VEN vs. DEC-VEN (CCR: 58% vs. 52%, P=0.66), but OS and EFS were longer for AZA-VEN (m-OS: 12.3 vs. 2.2 months, P<0.05; m-EFS: 9.2 vs. 2.1 months, P<0.05). Our analysis showed that combining VEN with AZA in newly diagnosed AML patients improved outcomes, but combining VEN with DEC did not. AZA-VEN was associated with improved outcomes compared to DEC-VEN. Further studies are needed to test the benefit of combining VEN with DEC.

## Introduction

Acute myeloid leukemia (AML) is a heterogeneous hematologic malignancy characterized by diverse cytogenetic and molecular abnormalities, including aberrant DNA methylation (1). The median age of patients with AML at diagnosis is 68 years (2). Older patients often respond poorly to cytotoxic chemotherapy due to adverse cytogenetic and molecular features (3), as well as comorbidities that render them vulnerable to toxicities (4–6). Consequently, there has been a shift toward the use of targeted rather than cytotoxic therapies in older AML patients (6).

The hypomethylating agents (HMA) azacitidine (AZA) and decitabine (DEC) are used to treat AML patients unlikely to tolerate or respond to cytotoxic chemotherapy, recently in combination with the B-cell lymphoma-2 (Bcl-2) inhibitor venetoclax (VEN) (7). AZA and DEC both irreversibly bind to DNA methyltransferase-1, which leads to both DNA hypomethylation and induction of DNA damage (8). Bcl-2, which inhibits the intrinsic apoptosis pathway, is overexpressed in AML cells, and VEN induces apoptosis in leukemia cells dependent on BCL-2 for survival (9). VEN combinations with DEC, AZA, or low-dose cytarabine (LDAC) were studied in uncontrolled phase I/II trials in previously untreated AML patients, demonstrating improved response rates and overall survival (OS) compared to historical controls (10, 11). Based on these results, the United States Food and Drug Administration (FDA) approved VEN in 2018 in combination with the above agents for the treatment of newly diagnosed AML in patients 75 years or older or with comorbidities precluding the use of intensive chemotherapy. Subsequently, VEN with AZA was found to improve outcomes in elderly or unfit AML patients, compared to AZA alone, in a phase III randomized clinical trial, with complete remission (CR) rate 36.7% vs. 17.9% (P<0.001), composite CR [CCR; CR plus CR with incomplete hematologic recovery (CRi)] 66.4% vs. 28.3% (P<0.001) and median OS (m-OS) 14.7 vs. 9.6 months (P<0.001) (11).

There is a deficiency of data on real-world outcomes of adding VEN to HMAs. Moreover, there are no studies comparing AZA-VEN with DEC-VEN. This propensity score-adjusted cohort study aims to evaluate CCR, OS and event-free survival (EFS) in newly diagnosed AML patients treated with HMAs with or without VEN.

## Methods

### Study Design and Comparison Groups

We conducted a single-center retrospective cohort study to compare CCR rates, OS, and EFS in adults (≥18 years old) with newly diagnosed AML treated with an HMA (AZA or DEC) with or without VEN from 2013 through 2020. Comparison groups included HMA vs. HMA-VEN, AZA vs. AZA-VEN, DEC vs. DEC-VEN, AZA-VEN vs. DEC-VEN and DEC vs. AZA. AZA was administered at a dose of 75 mg/m^2^ body surface area subcutaneously or intravenously on days 1-7 of 28-day cycles. DEC was administered at a dose of 20 or 10 mg/m^2^ body surface area intravenously on days 1-5 or 1-10 of 28-day cycles (12). Patients who received DEC 20 mg/m2 IV for 10 days of 28-day cycles were continued on this schedule until leukemia-free marrow was documented, after which they received DEC for 5 days of each 28-day cycle. VEN was given orally in a three-day ramp-up (100 mg, then 200 mg, then 400 mg), then 400 mg daily for a total of 28 days. Treatment could be initiated in inpatient or outpatient settings, depending on medical and social considerations. The choice of HMA (AZA vs. DEC) and duration of DEC (5 or 10 days) was based on the treating physician’s decision. Antimicrobial prophylaxis was also at the discretion of the treating physician. Azole antifungals were generally avoided during the first courses of venetoclax-containing regimens to give full-dose venetoclax.

Bone marrow aspirate and biopsy were obtained to assess response at the end of cycle one (~ Day 28). Treatment response was defined according to the 2017 European LeukemiaNet (ELN) criteria (13). Cycle two was initiated in patients with leukemia-free marrows with adequate count recovery or with persistent AML. In patients with pesistent disease, repeat bone marrow aspirate and biopsy were obtained at the end of cycle 2 or 3, based on the treating physician’s decision. Treatment was continued in the absence of disease progression or inability to tolerate therapy. The schedule of venetoclax could be altered in patients with poor count recovery or recurrent infections. In responding patients, bone marrow aspirate and biopsy were repeated for concern for relapse. CCR rate included CR+CRi and recorded at best response. OS was defined as the time from treatment initiation to death from any cause. EFS was defined from treatment initiation to induction failure, relapse, or death from any cause. Time to response (CR or CRi) was defined from treatment initiation to confirmation of response based on blood counts and bone marrow aspirate and biopsy findings. Treatment-related mortality (TRM) was assessed using 30-day and 60-day mortality from treatment initiation as surrogates (14). We also recorded causes of death (15). The study was approved by the University of Maryland Baltimore Institutional Review Board.

### Data Collection

Data were collected from medical records of all patients diagnosed with AML at the University of Maryland Greenebaum Comprehensive Cancer Center (UMGCCC) between 2013 and 2020.

Data collected included age, gender, ethnicity, Eastern Cooperative Oncology Group (ECOG) performance status, baseline comorbidities, AML categories (*de novo*, myelodysplasia-related, myeloproliferative-related, therapy-related), cytogenetics, myeloid mutations including *FLT3*, *IDH1*, *IDH2*, *NPM1*, *TP53, ASXL1 and RUNX1*, and treatments received, including transplant. Cytogenetic risk groups were defined using ELN 2017 criteria (13). Data were managed using the Research Electronic Data Capture (REDCap) electronic data capture tools hosted at the University of Maryland.

### Propensity Score Estimation

This study obtained the Average Treatment Effect (ATE) (16). We included the following variables in the propensity score model: age at diagnosis, gender, ethnicity, comorbidities, ECOG performance status, AML category, cytogenetic risk group at diagnosis, *FLT3*, *IDH1*, *IDH2*, *TP53, ASXL1*, and *RUNX1* mutation status, and previous HMA treatment. Different methods for matching were attempted, including 1:1 nearest neighbor, 1:2 nearest neighbor, full-matching, inverse probability weighting, and weighting by the odds. The method of matching/weighting was selected to achieve the lowest standardized biases differences, the smallest coefficients of variations, and the largest effective sample sizes. Weights were estimated using binary regression, generalized boosted modeling, covariate balancing or non-parametric covariate balancing. Weights obtained from full-matching or weighting were used to adjust outcomes. No patients were dropped in the full-matching/weighting process. A standardized bias score less than 0.25 was used as a criterion for adequate balancing (16). We used balance tables and Love plots to assess for covariate balance before and after matching.

### Statistical Analysis

Descriptive statistics were used for baseline characteristics. Categorical variables were presented as absolute numbers and percentages. Continuous variables were presented as means with standard deviations or medians with interquartile ranges (IQR). Baseline characteristics were compared using Pearson chi-square or Fisher’s exact test for categorical variables or t-test or ANOVA for continuous variables. OS, EFS and time to achieve CCR were compared using log-rank and Gehan Breslow-Wilcoxon rank tests. Both 30-day and 60-day mortality was compared between groups using Pearson chi-square or Fisher’s exact test. Multivariable and univariable Cox proportional hazards models were used to assess relative mortality. In addition, weighted regression models were obtained to estimate outcomes (17, 18). Multivariable logistic regression was used to assess predictors of 60-day mortality. To determine if *NPM1* mutational status modifies the effect of VEN combined with HMA on OS and EFS, we fitted a weighted Cox proportional hazards model with an interaction term between *NPM1* mutational status and treatment received. Regression diagnostics were used to evaluate model assumptions. Cluster-robust standard errors were used to account for subclass membership in the matching process. All statistical tests were two-sided, and P-values <0.05 were considered statistically significant. The R statistical packages’MatchIt’ and ‘WeightIt’ were used for propensity score modeling (19, 20). R-statistical software (version 4.1.1) was used for statistical analyses.

## Results

### Overview

#### Cohort Characteristics

We identified a total of 468 AML patients treated at UMGCCC during the study period (2013-2020) and included 170 patients who were treated with HMA or HMA with VEN as first-line therapy in this study. Median age was 71.5 years (IQR 63.4-78.6) and 99 (58%) were male. Patients included 35 treated with AZA alone (20.5%), 84 with DEC alone (49%), 24 with AZA-VEN (14.1%), and 27 with DEC-VEN (15.9%). Median follow-up for all patients in the study was 53 months (IQR 24.23-78.7). **Table 1** shows unadjusted baseline characteristics of all treatment groups.

**Table 1:** Baseline characteristics of newly diagnosed AML patients treated with azacitidine or decitabine with or without venetoclax.

	Azacitidine	Percentage/SD/IQR	Decitabine	Percentage/SD/IQR	Azacitidine plus Venetoclax	Percentage/SD/IQR	Decitabine plus Venetoclax	Percentage/SD/IQR	P-value
**Number**	35	–	84	–	24	–	27	–	–
**Female**	19	54.3	31	36.9	12.00	50.0	9.00	33.3	0.210
**Ethnicity**									0.101
** Causian**	25	71.4	65	77.4	14.00	58.3	24.00	88.9	
** Other**	9	25.7	19	22.6	10.00	41.7	3.00	11.1	
** Unknown**	1	2.9	0	0.0	0.00	0.0	0.00	0.0	
**Comorbidities**									
** Cardiovascular disease**	14	40.0	25	29.8	5.00	20.8	11.00	40.7	0.319
** Diabetes mellitus**	5	14.3	18	21.4	8.00	33.3	8.00	29.6	0.291
** Hypertension**	15	42.9	46	54.8	12.00	50.0	12.00	44.4	0.613
** CKD stage III-V/ESRD**	2	5.7	3	3.6	2.00	8.3	3.00	11.1	0.493
** Active Cancer**	3	8.6	7	8.3	1.00	4.2	0.00	0.0	0.421
**AML type**									0.708
** AML, *de novo***	17	48.6	36	42.9	12.00	50.0	11.00	40.7	
** AML with MDS/CMML changes**	10	28.6	27	32.1	5.00	20.8	12.00	44.4	
** AML with MPN**	1	2.9	8	9.5	3.00	12.5	1.00	3.7	
** Therapy-Related AML**	7	20.0	13	15.5	4.00	16.7	3.00	11.1	
**ELN 2017 Cytogenetic Category**									0.414
** Favorable Risk**	1	2.9	0	0.0	1.00	4.2	1.00	3.7	
** Intermediate Risk**	23	65.7	67	79.8	19.00	79.2	23.00	85.2	
** Unfavorable Risk**	2	5.7	3	3.6	2.00	8.3	1.00	3.7	
** Not performed/Poor banding, Inadequate**	9	25.7	14	16.7	2.00	8.3	2.00	7.4	
***FLT3*-ITD status**									0.187
***FLT3*-ITD mutated 1-49%**	4	11.4	8	9.5	1.00	4.2	1.00	3.7	
***FLT3*-ITD mutated 50-100%**	0	0.0	6	7.1	1.00	4.2	1.00	3.7	
**Not tested**	10	28.6	15	17.9	1.00	4.2	3.00	11.1	
***FLT3* WT**	21	60.0	55	65.5	21.00	87.5	22.00	81.5	
***FLT3*-TKD status**									0.274
***FLT3*-TKD mutated**	4	11.4	10	11.9	3.00	12.5	5.00	18.5	
**Not tested**	10	28.6	15	17.9	1.00	4.2	3.00	11.1	
***FLT3* WT**	21	60.0	59	70.2	20.00	83.3	19.00	70.4	
***NPM1* status**									0.14
***NPM1* mutated**	5	14.3	17	20.2	2	8.3	6	22.2	
**Not tested**	13	37.1	39	46.4	7	29.2	7	25.9	
***NPM1* WT**	17	48.6	28	33.3	15	62.5	14	51.9	
***TP53* status**									0.347
***TP53* mutated**	6	17.1	11	13.1	3.00	12.5	7.00	25.9	
**Not tested**	13	37.1	39	46.4	7.00	29.2	7.00	25.9	
***TP53* WT**	16	45.7	34	40.5	14.00	58.3	13.00	48.1	
***RUNX1* status**									0.220
***RUNX1* mutated**	3	8.6	6	7.1	5.00	20.8	5.00	18.5	
**Not tested**	13	37.1	39	46.4	7.00	29.2	7.00	25.9	
***RUNX1* WT**	19	54.3	39	46.4	12.00	50.0	15.00	55.6	
***ASXL1* status**									0.132
***ASXL1* mutated**	2	5.7	3	3.6	4.00	16.7	4.00	14.8	
**Not tested**	7	20.0	39	46.4	7.00	29.2	7.00	25.9	
***ASXL1* WT**	20	57.1	42	50.0	13.00	54.2	16.00	59.3	
									0.679
**ECOG status III/IV**	4	11.4	14	16.7	2.00	8.3	5.00	18.5	
**ECOG status Unknown**	3	8.6	2	2.4	0.00	0.0	0.00	0.0	
**Previous HMA**	1	2.9	2	2.4	0.00	0.0	5.00	18.5	0.003
**Year of treatment initiation**									<0.001
**2013-2016**	13	37.1	51	60.7	0	0	2	7.4	
**2017-2018**	15	42.9	25	29.8	2	8.3	4	14.8	
**2019-2020**	7	20	8	9.5	22	91.7	21	77.8	
**Age (Average ± SD)**	68.5	14.4	70.3	10.7	69.7	12.2	71.1	11.6	0.871
**Age (Median, IQR)**	69.3	60.9-77.7	71.9	65.2-78.8	70.5	67.2-78.4	72.6	64.7-79	0.704

HMA, hypomethylating agent; SD, standard deviation; IQR, interquartile range; CKD, chronic kidney disease; ESRD, end-stage renal disease; AML, acute myeloid leukemia; MDS, myelodysplastic syndrome; CMML, chronic myelomonocytic leukemia; MPN, myeloproliferative neoplasm; ELN, European LeukemiaNet; ITD, internal tandem duplication; TKD, tyrosine kinase domain; WT, wild type.

#### Unadjusted Cohort Outcomes: CCR, OS, EFS and TRM

**Figure 1:**
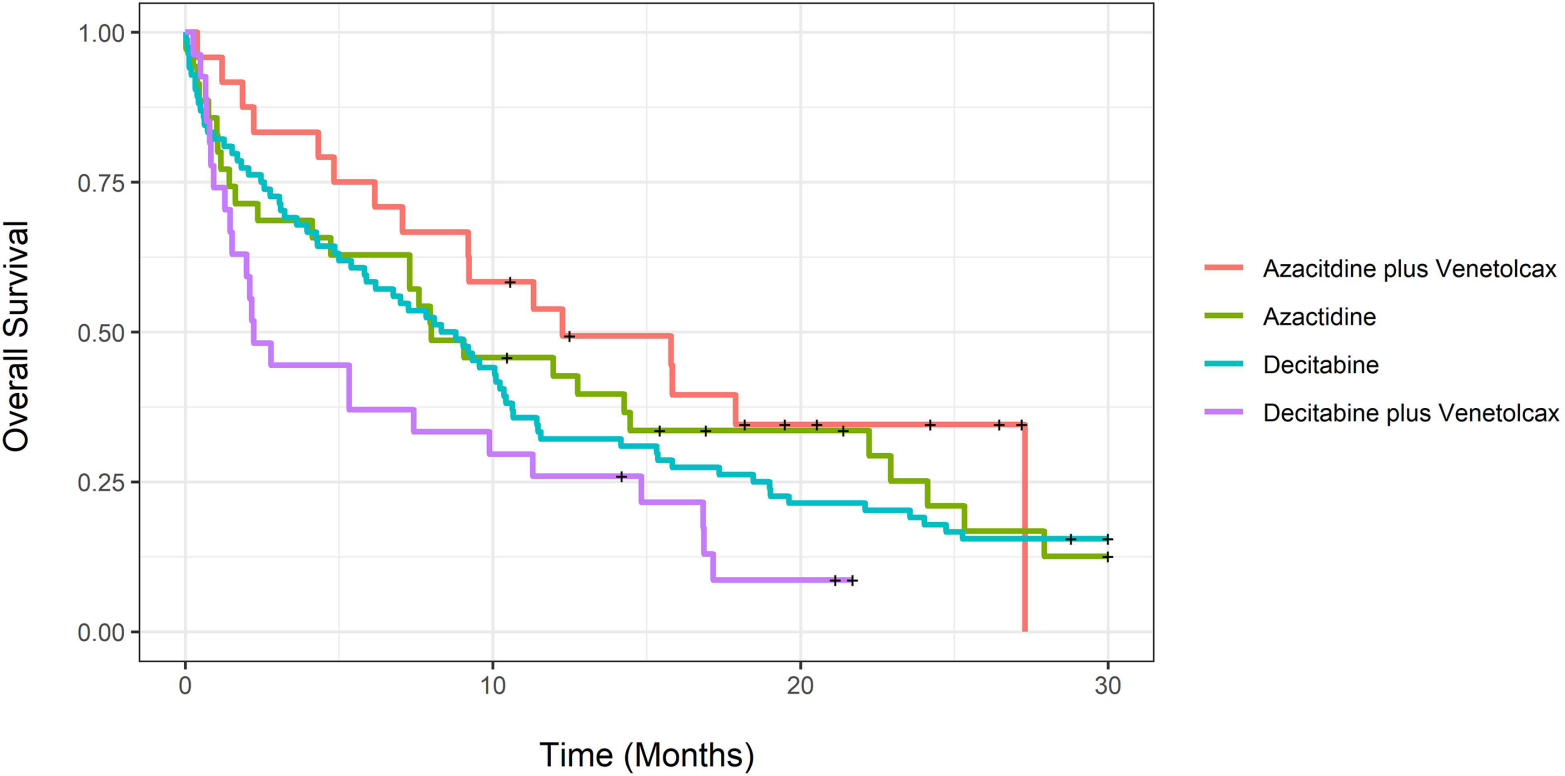
Unadjusted overall survival for newly diagnosed acute myeloid leukemia patients treated with azacitidine with venetoclax, azacitidine, decitabine with venetoclax or decitabine.

**Figure 2:**
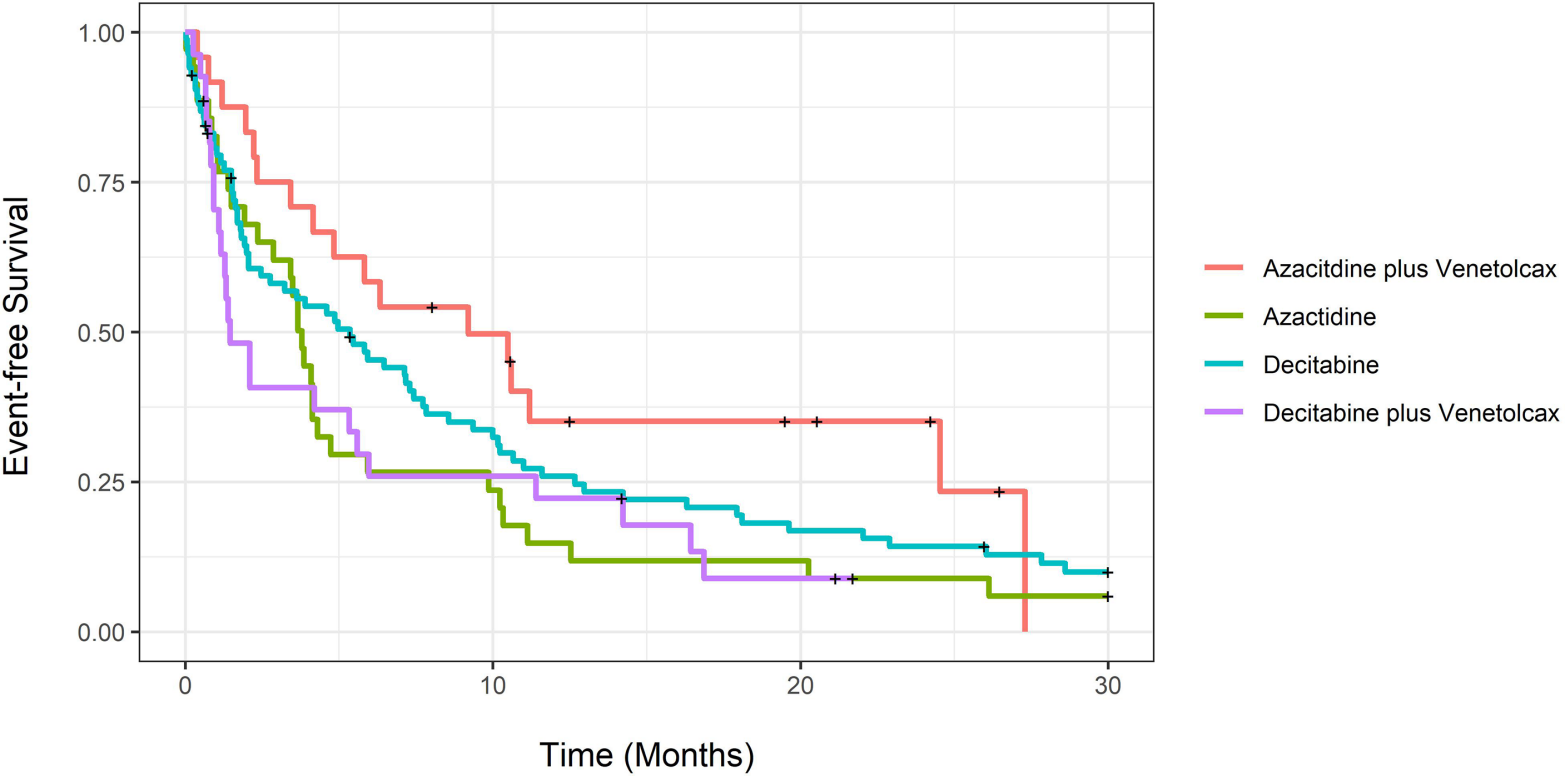
Unadjusted event-free survival for newly diagnosed acute myeloid leukemia patients treated with azacitidine with venetoclax, azacitidine, decitabine with venetoclax or decitabine.

Unadjusted CCR rates were 58.3% for patients treated with AZA-VEN, 14.3% for AZA, 44.4% for DEC-VEN, and 33.3% for DEC (P=0.93). We further tested the median time to achieve CCR; it was 2.97 months (CI: 1.3-NA) for AZA-VEN, 6.5 months (CI: 4.3-NA) for AZA, 2.57 months (CI: 1.33-NA) for DEC-VEN, and 4.97 months (3.07-16.6) for DEC (P=0.003). **Figure 1** demonstrates unadjusted overall survival curves and **Figure 2** demonstrates unadjusted event-free survival curves for all treatment arms. We subsequently determined the causes of death in each arm (**eTable 1**). **eTable 2** describes predictors of 60-day mortality using multivariable logistic regression. Both mutated *TP53* and ECOG stage III-IV were associated with increased 60-day mortality. In contrast, age was not predictive. An increase of age by one year was associated with an absolute increase of 2.5% odds ratio of 60-day mortality, adjusted for other variables; however, this was not statistically significant. We also estimated whether median age (71 years) would predict 60-day mortality; this was not statistically significant.

### HMA-VEN vs. HMA

#### Baseline Characteristics and Follow-Up

Unadjusted and propensity score-adjusted baseline characteristics of patients treated with HMA-VEN vs. HMA are shown in **Table 2**. After matching, there were no statistically significant differences in baseline characteristics between the two groups. Covariate balance before and after propensity score weighting is shown in **eFigure 1**. Median follow-up by reverse Kaplan-Meier for patients treated with HMA and HMA-VEN was 78.6 months (CI: 53.03; not calculable (NC)) compared to 21.13 (CI: 19.50;26.47).

**Table 2:** Unadjusted and Adjusted baseline characteristics of newly diagnosed AML patients treated with HMA with or without venetoclax.

	Unadjusted					Adjusted				
	HMA	Percentage/SD/IQR	HMA-VEN	Percentage/SD/IQR	P-value	HMA^β^	Percentage/SD/IQR	HMA-VEN^β^	Percentage/SD/IQR	P-value
**Number**	119	–	51	–		110	–	38	–	–
**Female**	50	42.0	21	41.2	1.000^α^	43	39.0	15	40.0	0.955
**Ethnicity**					0.782					0.749
**Causian**	90	75.6	38	74.5		83	75.0	29	75.0	
**Other**	28	23.5	13	25.5		26	24.0	10	25.0	
**Unknown**	1	0.8	0	0.0		1	0.6	0	0.0	
**Comorbidities**										
**Cardiovascular disease**	39	32.8	16	31.4	1.000^α^	37	34.0	13	34.0	0.902
**Diabetes mellitus**	23	19.3	16	31.4	0.130	23	21.0	11	28.0	0.346
**Hypertension**	61	51.3	24	47.1	0.738	52	47.0	16	42.0	0.580
**CKD stage III-V/ESRD**	5	4.2	5	9.8	0.286	6	5.0	3	8.0	0.540
**Active Cancer**	10	8.4	1	2.0	0.221	8	7.0	2	6.0	0.859
**AML type**					0.965					0.964
**AML, *de novo***	53	44.5	23	45.1		50	45.3	16	43.0	
**AML with MDS/CMML changes**	37	31.1	17	33.3		34	30.8	12	31.0	
**AML with MPN**	9	7.6	4	7.8		8	7.6	2	6.0	
**Therapy-Related AML**	20	16.8	7	13.7		18	16.2	7	19.0	
**ELN 2017 Cytogenetic Category**					0.151					0.960
**Favorable Risk**	1	0.8	2	3.9		2	1.7	1	2.0	
**Intermediate Risk**	90	75.6	42	82.4		85	77.5	31	80.8	
**Unfavorable Risk**	23	19.3	4	7.8		18	16.2	5	13.0	
**Not performed/Poor banding, Inadequate**	5	4.2	3	5.9		0	0.0	2	4.1	
***FLT3*-ITD status**					0.058					0.871
***FLT3*-ITD mutated 1-49%**	12	10.1	2	3.9		10	8.7	3	8.9	
***FLT3*-ITD mutated 50-100%**	6	5.0	2	3.9		6	5.2	2	6.2	
***FLT3* WT**	25	21.0	4	7.8		20	17.9	4	11.6	
**Not tested**	76	63.9	43	84.3		75	68.2	28	73.3	
***FLT3*-TKD status**					0.106					0.662
***FLT3*-TKD mutated**	14	11.8	8	15.7		14	12.7	5	13.1	
***FLT3* WT**	25	21.0	4	7.8		20	17.9	4	11.7	
**Not tested**	80	67.2	39	76.5		76	69.4	29	75.2	
***NPM1* status**					0.062					0.86
***NPM1* mutated**	22	18.5	8	15.7		32	18.5	22	15.2	
***NPM1* WT**	45	37.8	29	56.9		72	41.6	67	46.2	
**Not tested**	52	43.7	14	27.5		69	39.9	56	38.6	
***TP53* status**					0.135					0.993
***TP53* mutated**	17	14.3	10	19.6		16	14.4	6	15.2	
***TP53* WT**	52	43.7	14	27.5		44	39.9	15	38.6	
**Not tested**	50	42.0	27	52.9		50	45.7	18	46.2	
***RUNX1* status**					0.028					0.958
***RUNX1* mutated**	9	7.6	10	19.6		11	10.4	4	11.8	
***RUNX1* WT**	52	43.7	14	27.5		44	39.9	15	38.9	
**Not tested**	58	48.7	27	52.9		55	49.7	19	49.3	
***ASXL1* status**					0.013					0.913
***ASXL1* mutated**	5	4.2	8	15.7		8	6.9	3	8.3	
***ASXL1* WT**	52	43.7	14	27.5		44	39.7	15	38.9	
**Not tested**	62	52.1	29	56.9		59	53.4	20	52.8	
**ECOG status III/IV**	18	15.1	7	13.7	0.915	16	14.9	6	14.5	0.943
**Previous HMA**	3	2.5	5	9.8	0.097	3	2.9	2	4.1	0.673
**Age (Average ± SD)**	69.79	11.9	70.46	11.8	0.736	69.9	4.6	69.2	6.9	0.736
**Age (Median, IQR)**	71.4	63-78.3	71.9	65.6-78.6	0.867	71.4	62.9-79.05	69.6	64.4-78.14	0.511

HMA, hypomethylating agent; SD, standard deviation; IQR, interquartile range; CKD, chronic kidney disease; ESRD, end-stage renal disease; AML, acute myeloid leukemia; MDS, myelodysplastic syndrome; CMML, chronic myelomonocytic leukemia; MPN, myeloproliferative neoplasm; ELN, European LeukemiaNet; ITD, internal tandem duplication; TKD, tyrosine kinase domain; WT, wild type. ^α^Approximated. ^β^Weighted sample size. N.B. No patients dropped from the analysis.

#### CCR, OS and EFS

**Figure 3:**
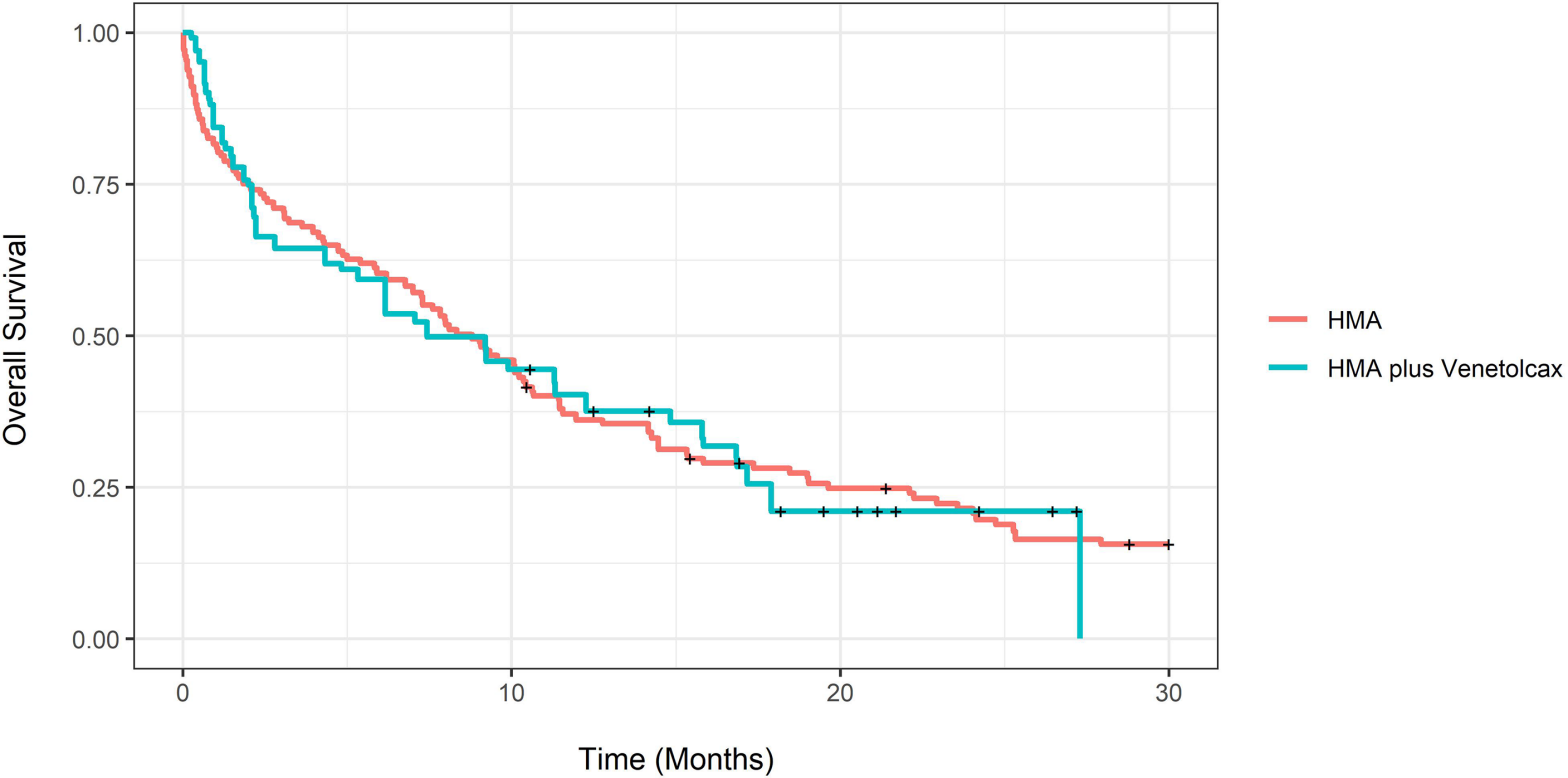
Propensity score-adjusted overall survival for newly diagnosed acute myeloid leukemia patients treated with hypomethylating (HMA) vs. HMA plus venetoclax.

**Figure 4:**
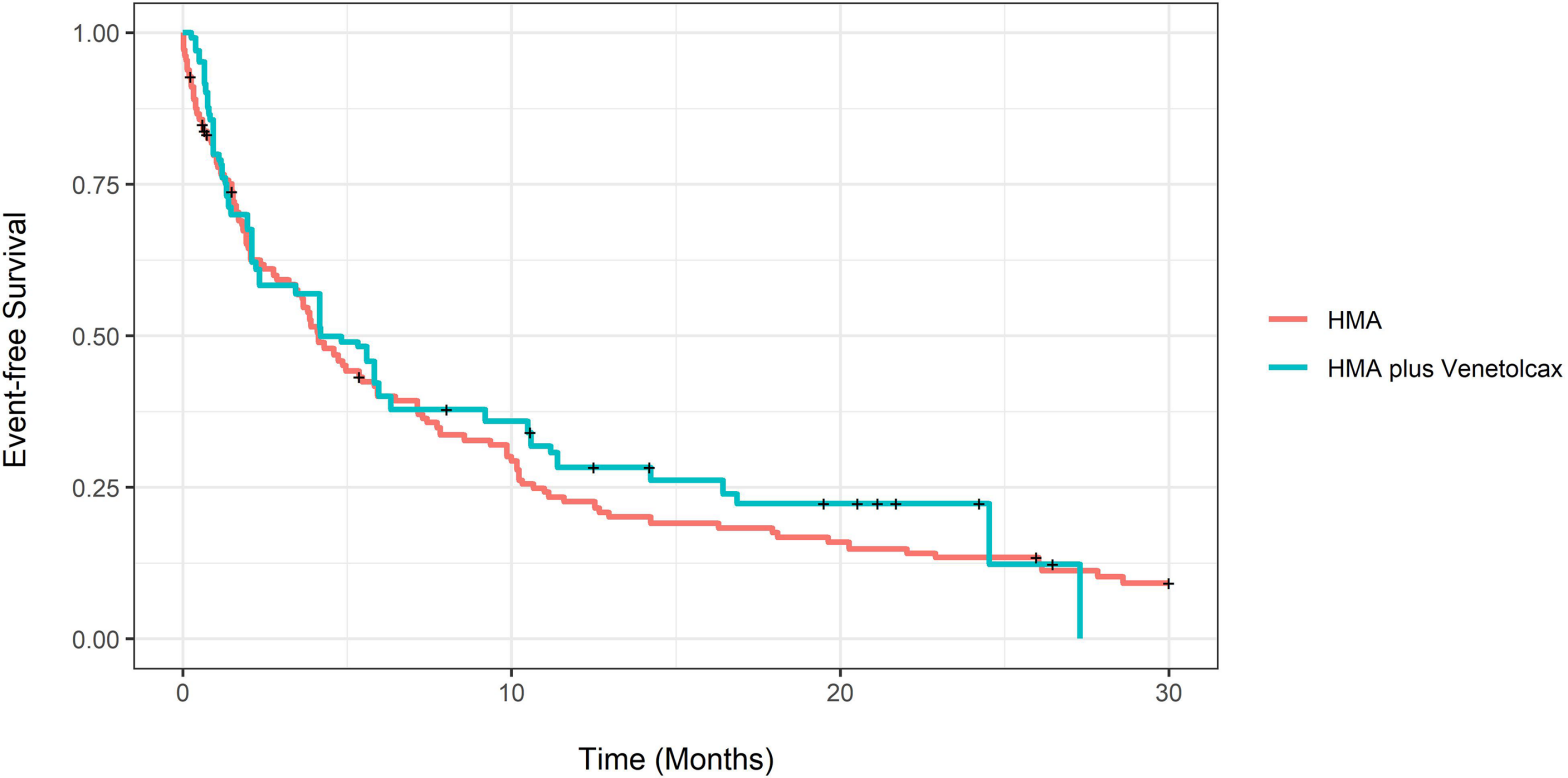
Propensity score-adjusted event-free survival of newly diagnosed acute myeloid leukemia patients treated with hypomethylating (HMA) vs. HMA plus venetoclax.

The adjusted CCR rate for patients treated with HMA-VEN and HMA was 52% compared to 27% (P=0.004). In contrast, adjusted m-OS was 7.43 (CI:4.33-16.8) compared to 8.8 (CI: 6.77-11.4) months (log-rank P= 0.94), and the unadjusted m-OS was 7.43 months (CI: 4.33-15.8) compared to 8.3 months (CI: 6.77-10.6) (log-rank P=0.8). Adjusted OS at months 12 and 24 for patients treated with HMA with and without VEN was not statistically different (**eTable 3**; **Figure 3**). On weighted-univariable Cox proportional hazards regression, the relative mortality for HMA-VEN compared to HMA was not statistically different [HR (hazard ratio): 1.01, CI: 0.67-1.53, P= 0.94]. The adjusted m-EFS for patients treated with HMA-VEN vs. HMA was 4.2 months (CI: 3.23-6.47) compared to 4.13 (CI: 2.1-11.4) (log-rank P=0.54). Adjusted EFS at months 12 and 24 for patients on HMA-VEN and HMA was not statistically different (**eTable 4**; **Figure 4**). Additionally, the relative mortality or relapse rate was not different in patients treated with HMA-VEN compared to HMA (HR: 0.87, CI: 0.58-1.33, P=0.54). The adjusted 12-month transplant rate for patients treated with HMA+VEN vs. HMA was 10.6% (CI: 0%-23.8%) vs. 18.3% (CI: 4.9%-29.8%) (P=0.32).

To determine if *NPM1* mutational status modifies the effect of combining VEN with HMA, an interaction term was modeled in a weighted Cox proportional hazards regression. The impact of VEN on OS or EFS did not differ in *NPM1* mutated AML compared to wild-type (HR: 1.04, CI: 0.24-4.52 P=0.96), (HR: 0.91, CI: 0.42-1.95, P=0.9).

### AZA-VEN vs. AZA

#### Baseline Characteristics and Follow-Up

The adjusted median age for AZA-VEN vs. AZA was 69.2 (IQR:56.5-78.13) vs. 71.2 (IQR: 60.85-77.6) years (P=0.81). Median follow-up was 20.53 (CI: 18.20-26.47) vs. 53.03 (CI: 21.40-NC) months. Covariate balance before and after propensity score weighting is shown in **eFigure 2**.

#### CCR, OS, EFS and TRM

The adjusted CCR rate for patients treated with AZA-VEN and AZA was 54% compared to 10% (P=0.0007), and the adjusted m-EFS was 10.5 (CI: 5.83-NC) compared to 3.8 (CI: 3.43-5.93) months (P=0.023) (**eFigure 3**). The adjusted-relative mortality or relapse rate was lower in patients treated with AZA-VEN compared to AZA (HR: 0.49, CI: 0.28-0.95, P=0.034), but adjusted EFS at 12 and 24 months for patients on AZA-VEN and AZA was not statistically different (**eTable 5**).

In contrast, the adjusted m-OS for patients treated with AZA-VEN compared to AZA and was 17.9 (CI: 11.3-NC) vs. 8 (CI: 7.3-24.1) months (P= 0.263), and the unadjusted m-OS was 12.3 (CI: 9.2-NC) vs. 8 (CI: 4.73-22.9) months (P=0.4). Adjusted OS difference at 12 and 24 months for patients treated with AZA-VEN vs. AZA was not statistically different(**eTable 6**), and adjusted-relative mortality did not differ significantly in patients treated with AZA-VEN compared to AZA (HR: 0.71, CI: 0.37-1.31, P= 0.26). **eFigure 4** demonstrates adjusted OS for patients treated with AZA-VEN vs. AZA.

To explore if VEN addition to AZA increases toxicity, we estimated the unadjusted and adjusted TRM. Unadjusted and adjusted 30-day TRM in AZA-VEN vs. AZA was 4.2% vs. 14.4% (P=0.41) and 2% vs. 13.6% (P=0.07), respectively. Unadjusted and adjusted 60-day TRM in AZA-VEN compared to AZA was 12.5% vs. 28.6% (P=0.25) and 11.8% vs. 22.4% (P=0.35), respectively.

### DEC-VEN vs. DEC

#### Baseline Characteristics and Follow-Up

The adjusted median age for DEC-VEN vs. DEC was 72.6 (IQR: 64.36-77.89) compared to 71.9 (IQR: 64.4-79.05) years (P=0.75). Median follow-up for patients treated with DEC-VEN and DEC was 21.13 (CI: 21.13-NC) compared to 78.70 (CI: 53.03-NC) months. Covariate balance before and after propensity score weighting is shown in **eFigure 5**. On the DEC only arm, 55 patients received 20 mg/m^2^ for 10 days (65%), 21 received 10 mg/m^2^ for 10 days (25%), 7 received 20 mg/m^2^ for 5 days (8%) and one received 10 mg/m^2^ for 5 days. On the DEC-VEN arm, 24 patients received 20 mg/m^2^ for 10 days (89%) and 3 received 20 mg/m^2^ for 5 days (11%) (**eTable 7**).

#### CCR, OS, EFS and TRM

The adjusted CCR rate for patients treated with DEC-VEN and DEC was 43% compared to 32% (P=0.35). The adjusted m-OS for patients was 5.33 (CI: 1.53-11.3) compared to 8.3 (CI: 5.83-10.4) (P=0.076) months, and unadjusted m-OS 2.23 (CI: 1.53-11.3) and 8.57 (CI: 5.83-10.6) months (P=0.08). Adjusted OS difference at 12 months was not statistically different (**eTable 8**). Adjusted-relative mortality did not differ significantly between patients treated with DEC-VEN compared to DEC (HR: 1.47 CI: 0.94-2.29, P= 0.08) (**eFigure 6**). Adjusted m-EFS for patients with DEC-VEN vs. DEC was 2.1 (CI: 1.17-5.97) compared to 4.97 (CI: 2.07-7.73) months (P=0.16). Adjusted EFS at 12 months was not statistically different (**eTable 9**), and adjusted-relative mortality or relapse was not different in patients treated with DEC-VEN compared to DEC (HR: 1.37, CI: 0.88-2.15, P= 0.16) (**eFigure 7**).

TRM was 25.9% vs. 17.9% (P=0.52) and 24.2% vs. 18.6% (P=0.53) in DEC-VEN vs. DEC arms, respectively. Unadjusted and adjusted 60-day TRM in DEC-VEN compared to DEC was 40.7% vs. 22.6% (P=0.11) and 37.1% vs. 23.5% (P=0.17), respectively.

#### Subgroup Analysis: Ten-Day DEC 20 mg/m^2^-VEN vs. DEC 20 mg/m^2^ Alone

In this subgroup analysis, we compared 24 patients who received DEC 20 mg/m^2^ for 10 days combined with VEN to 55 patients who received DEC 20 mg/m^2^ for 10 days alone. The adjusted CCR rate was 54% compared to 34% (P=0.15), and the adjusted m-OS was 2.17 months (CI: 1.47-16.8) compared to 8.8 months (CI: 6.2-10.4) (P=0.38) and adjusted m-EFS 1.47 (CI: 1.17-16.43) compared to 5.37 (CI: 2.07-7.43) months (P=0.73).

### AZA vs. DEC

#### Baseline Characteristics and Follow-Up

The adjusted median age for AZA vs. DEC was 71.5 (IQR: 61-86) compared to 71.95 (IQR: 65.3-77.9) years (P=0.74). Median follow-up for patients treated with AZA and DEC was 53.03 (CI: 21.40-NC) compared to 78.70 (CI: 66.10-NC) months. Covariate balance before and after propensity score weighting is shown in **eFigure 8**.

#### CCR, OS, EFS and TRM

The adjusted CCR rate for patients treated with AZA vs. DEC was 13% compared to 33% (P=0.04), but the adjusted m-OS for patients treated with AZA vs. DEC was 9.07 months (CI: 7.3-25.3) compared to 9.03 months (CI: 6.2-10.6) (P= 0.63). The unadjusted m-OS for patients treated with AZA and DEC was 8.0 (CI: 4.73-22.9) and 8.57 (CI: 5.83-10.6) months (P=0.6). Adjusted OS difference at years 1-4 for patients with AZA vs. DEC was not significantly different (**eTable 10**). Adjusted-relative mortality for patients treated with DEC compared to AZA was also not significantly different (HR: 1.25, CI: 0.77-2.06, P= 0.36). **eFigure 9** demonstrates adjusted OS. The adjusted m-EFS for patients treated with AZA vs. DEC was 3.8 (CI: 3.5-9.87) compared to 5.47 (CI: 2.77-7.83) months (P=0.63) (**eFigure 10**). Adjusted EFS at years 1-4 for patients treated with AZA vs. DEC was not different (**eTable 11**).The adjusted-relative mortality or relapse rate for patients treated DEC compared to AZA was not different (HR: 0.8, CI: 0.52-1.24, P= 0.32).

The unadjusted and adjusted 30-day TRM in AZA compared to DEC was 14.3% vs. 17.9% (P=0.84) and 12.5% vs. 15.1% (P=0.66), respectively. The unadjusted and adjusted 60-day TRM in AZA compared to DEC was 28.6% vs. 22.6% (P=0.65) and 20.5% vs. 19.3% (P=0.87), respectively.

### AZA-VEN vs. DEC-VEN

#### Baseline Characteristics and Follow-Up

The adjusted median age for AZA-VEN vs. DEC-VEN was 69.9 (IQR: 67.01-80.04) compared to 73.02 (IQR: 64.37-79.8) years (P=0.60). Median follow-up did not differ, at 20.53 (CI: 18.20-26.47) compared to 21.13 (CI: 21.13-NC) months. Covariate balance before and after propensity score weighting is shown in **eFigure 11**.

#### CCR, OS, EFS and TRM

The adjusted CCR rate for patients treated with AZA-VEN vs. DEC-VEN did not differ, at 58% compared to 52% (P=0.66), but adjusted m-OS was 12.3 (CI: 9.2-NC) compared to 2.8 (CI: 2-16.8) months (P= 0.0056), and unadjusted m-OS was 12.27 (CI: 9.2-NC) compared to 2.23 (CI: 1.53-11.3) months (P=0.009). Adjusted OS at 12 months was not different (**eTable 12**; **eFigure 12**). The adjusted-relative mortality was higher in patients treated with DEC-VEN compared to AZA-VEN (HR 2.22, CI: 1.16-4.26, P= 0.016). Adjusted m-EFS for patients with AZA-VEN vs. DEC-VEN was 9.2 (CI: 4.83-NC) compared to 2.1 (CI: 1.4-16.4) months (P=0.035). Adjusted EFS at twelve months was not different(**eTable 13**; **eFigure 13**). The adjusted-relative mortality or relapse was higher for DEC-VEN compared to AZA-VEN (2.02, CI: 1.04-3.92, P= 0.0374).

Unadjusted and adjusted 30-day TRM in DEC-VEN compared to AZA-VEN was 25.9% vs. 4.2% (P=0.08) and 21.4% vs 2.4% (P=0.03), respectively. Unadjusted and adjusted 60-day TRM in DEC-VEN compared to AZA-VEN was 40.7% vs. 12.5% (P=0.052) and 37.2% vs. 11.6% (P=0.04), respectively.

## Discussion

There is a paucity of studies reporting real-world treatment outcomes of AZA-VEN compared to AZA alone and AZA-VEN compared to DEC-VEN in patients with newly diagnosed AML. Moreover, we used causal-inferential methods to compare outcomes of the treatment groups. **eTable 14** summarizes studies investigating the outcomes of combining VEN with HMAs. Consistent with the Mayo Clinic report (21), the addition of VEN to HMA did not improve CCR, OS, or EFS compared to HMA alone. This is likely due to the different effects of combining VEN with AZA compared to DEC, as shown in our analysis. In the subgroup analysis, combining VEN with AZA resulted in a more than two-fold increase in CCR, m-OS, and m-EFS, though the improvement in OS was not statistically significant. These data were consistent with the phase III VIALE-A trial outcomes comparing AZA-VEN and AZA (22). In contrast, the addition of VEN to DEC resulted in a higher CCR but a nonsignificant lower m-OS and m-EFS compared to DEC. The higher CCR indicates improved leukemia control, but the lower OS in DEC-VEN might be explained by increased TRM. Both 30-day and 60-day mortality was numerically higher in DEC-VEN compared to DEC alone but differences were not statistically significant. Early death due to infection was two times higher in DEC-VEN compared to DEC, but the difference was also not statistically significant. Progressive disease and relapse were responsible for approximately half of deaths in both DEC-VEN and DEC arms. As a sensitivity analysis, we compared patients who received 20 mg/m^2^ DEC for 10 days with VEN to patients who received 20 mg/m^2^ DEC for 10 days; the outcomes were qualitatively similar to the combined 5- and 10-day DEC -VEN vs. DEC comparison.

Outcomes of DEC-VEN in newly diagnosed AML have only been investigated in single-arm studies. In a phase Ib trial, 72 newly diagnosed AML patients received DEC-VEN, with a CCR rate of 71% and m-OS of 14.2 months (11). The DEC dose used was 20 mg/m2 IV for 5 days. In a phase II trial, Maiti et al. reported the outcomes of combining VEN with DEC 20 mg/m2 IV for 10 days in 28-day cycles until CR, followed by 5-day cycles, in 219 patients with AML and high-risk myelodysplastic syndrome (MDS) and chronic myelomonocytic leukemia (CMML) (23). The CCR rate was 83% and m-OS 16.2 months. In our study, patients who received DEC-VEN primarily had *de novo* AML, intermediate-risk cytogenetics, and wild-type *TP53* (**Table 1**), which likely does not explain the inferior survival of patients in this group. Moreover, it is unlikely that differences in baseline characteristics between the DEC-VEN and DEC groups in our study explain the difference in outcomes, as standardized biases were all <0.25 post propensity score weighting. The FDA granted approval of VEN use in combination with DEC in unfit newly diagnosed AML patients despite the lack of any comparative studies of DEC-VEN with DEC.

In our study, DEC was associated with a higher CCR rate (33% vs. 13%) and a nonsignificant difference in PFS (5.5 vs. 3.8 months) compared to AZA, but OS was similar (9 vs. 9 months). This indicates improved leukemia control but greater toxicity with DEC, with a resultant similar OS. These outcomes were similar to those in previous reports (24, 25).

In our analysis, patients who received DEC-VEN had a similar CCR rate but worse OS and PFS than those treated with AZA-VEN. Because of the limited number of patients in this comparison, we could not adjust for some of the comorbidities or for *ASXL1* or *RUNX1* mutation status (**eFigure 11**). As a sensitivity analysis, we conducted weighted-multivariable Cox proportional hazards regression to account for unbalanced covariates, with no qualitative difference in outcomes compared to the above (results not shown). Patel et al. reported real-world experience with combining VEN with AZA (54 patients), DEC (52 patients), and LDAC (6 patients) (26). M-OS was 11.3, 13.9, and 6.5 months (P=0.77), respectively. The reported m-OS was higher than in our study. However, this study did not adjust for baseline characteristics and was reported as an abstract; hence awaiting final analysis is needed. Moreover, this study did not include HMA alone as a comparator arm.

In a retrospective study of 28, 47 and 228 *NPM1*-mutated AML patients treated with HMA-VEN, HMA, and induction chemotherapy, Lochowiez et al. found that HMA-VEN was associated with a 69% decrease in mortality compared to induction chemotherapy (27). On the other hand, HMA was associated with a 68% increased mortality compared to induction chemotherapy. However, the Cox proportional hazards model did not account for important confounders, including *TP53* mutational status or comorbidities. In our study, *NPM-1* mutational status did not predict or modify the effect of combining VEN with HMA on OS or EFS.

The major limitation of this study is that it is a retrospective single-center experience, which may limit its external generalizability. Moreover, the limited number of patients included in the study might lead to type II error. On the other hand, single-center experiences improve the homogeneity of important variables, including treating physicians, nurses, pharmacy staff, and other ancillary services. Observational studies are susceptible to confounding on observable and non-observable covariates. In our study, we used propensity score weighting to control for observable confounding and obtain causal associations. All of our covariates were well-balanced and achieved standardized biases scores of less than 0.25. Unlike most observational studies, we adjusted our outcomes to baseline comorbidities. Another limitation of our study is the variable doses of DEC used in the DEC and DEC-VEN arms. We conducted a subanalysis of 10-day DEC-VEN compared to 10-day DEC, as mentioned above.

One of the strengths of our study is the completeness of data with only 7% of patients lost to follow-up. OS in prospective studies is frequently censored at disease progression, leading to a potentially biased estimation of OS, which may differ from real-life experience. Multiple statistical tests increase the risk for type I error. Adjustment for multiple statistical testing is required for confirmatory randomized trials and when exploring several outcomes in the same comparison (28). To improve adjustment for baseline characteristics using weighting, we conducted head-to-head comparisons of each regimen rather than single weighted-statistical tests for all regimens. We used cluster-robust standard errors to decrease the risk for type I error. We determined CCR, EFS and OS in each individual comparison. Because our study is exploratory, its results need confirmation through multi-institutional collaboration. Year of treatment initiation varied between comparison arms, which could introduce bias because of variation in supportive care. Despite this potential bias, single-agent DEC was associated with a nonsignificant higher m-OS and m-EFS than DEC-VEN.

## Conclusion

Our propensity-score-adjusted cohort study showed that combining VEN with AZA in newly diagnosed AML patients resulted in improved outcomes, but that this benefit was not seen when VEN was combined with DEC. Randomized, controlled trials or multi-institutional studies comparing DEC-VEN with DEC with adjustment for baseline characteristics are needed to further define the efficacy and safety of combining VEN with DEC.

## Data Availability Statement

Data is available on reasonable request. Requests to access the datasets should be directed to Moaath Mustafa Ali, moaath_mustafa@yahoo.com.

## Ethics Statement 

The studies involving human participants were reviewed and approved by University of Maryland Medical Center IRB. Written informed consent for participation was not required for this study in accordance with the national legislation and the institutional requirements.

## Author Contributions

All authors had full access to all the data and analysis in the study and take responsibility for the integrity of data and the accuracy of the data analysis. Study conception and design: MMA, EC, and MB. Data Collection: EC, MMA, HA, and KK. Analysis and Interpretation: MMA, EC, HA, KK, JL, SL, SN, VD, MB, and AE. Draft manuscript preparation: EC, MMA, and MB. Statistical analysis: MMA. Critical Review of Manuscript: HA, KK, JL, SL, SN, VD, MB, and AE. Administrative and technical support: MMA. Supervision: MMA and MB. All authors contributed to the article and approved the submitted version.

## Funding

Supported by NIH-NCI grant P30 CA134274 and University of Maryland, Baltimore UMMG Cancer Research Grant #CH 649 CRF issued by the State of Maryland Department of Health and Mental Hygiene (DHMH) under the Cigarette Restitution Fund Program.

## Conflict of Interest

The authors declare that the research was conducted in the absence of any commercial or financial relationships that could be construed as a potential conflict of interest.

## Publisher’s Note

All claims expressed in this article are solely those of the authors and do not necessarily represent those of their affiliated organizations, or those of the publisher, the editors and the reviewers. Any product that may be evaluated in this article, or claim that may be made by its manufacturer, is not guaranteed or endorsed by the publisher.
