# Supplementary material for: Outcomes of Newly Diagnosed Acute Myeloid Leukemia Patients Treated With Hypomethylating Agents With or Without Venetoclax: A Propensity Score-Adjusted Cohort Study
Source: Front Oncol. 2022 Mar 31;12:858202. doi: 10.3389/fonc.2022.858202 (PMC9008336; doi:10.3389/fonc.2022.858202)
Supplement: Supplementary file 1 [file DataSheet_1.docx]

**Supplementary file for the article titled: “****Outcomes of Hypomethylating Agents with and without Venetoclax in Newly Diagnosed Acute Myeloid Leukemia Patients: A Propensity Score-Adjusted Cohort Study.”**

Contents

[Causes of death 2](#_Toc96864831)

[Treatment-related mortality predictors 3](#_Toc96864832)

[HMA vs. HMA with Venetoclax 4](#_Toc96864833)

[Azacitidine vs. Azacitidine with Venetoclax 6](#_Toc96864834)

[Decitabine vs. Decitabine with Venetoclax 11](#_Toc96864835)

[Azacitidine vs. Decitabine 17](#_Toc96864836)

[Azacitidine plus Venbtoclax vs. Decitabine plus Venetoclax 22](#_Toc96864837)

[Studies investigating the outcomes of combining Venetoclax with HMAs 27](#_Toc96864838)

[References: 30](#_Toc96864839)

# Causes of death

eTable 1 Causes of death in AML patients treated with hypomethylating agents with or without Venetoclax.

|  | Azacitdine plus Venetolcax | Percentage | Azacitidine | Percentage | Decitabine | Percentage | Decitabine plus Venetolcax | Percentage | P-value |
| --- | --- | --- | --- | --- | --- | --- | --- | --- | --- |
| Number of patients |  | 16 |  | 29 |  | 80 | 24 |  | 0.55 |
| Cardiovascular | 0 | 0 | 1 | 3.4 | 0 | 0 | 0 | 0 |  |
| Early death-Infection^α^ | 1 | 6.3 | 4 | 13.8 | 5 | 6.3 | 3 | 12.5 |  |
| Early death-Other^α^ | 0 | 0 | 1 | 3.4 | 9 | 11.3 | 4 | 16.7 |  |
| Hemorrhage | 0 | 0 | 1 | 3.4 | 1 | 1.3 | 0 | 0 |  |
| Infection | 1 | 6.3 | 3 | 10.3 | 2 | 2.5 | 2 | 8.3 |  |
| Organ failure | 0 | 0 | 2 | 6.9 | 4 | 5 | 0 | 0 |  |
| Other/Unknown | 3 | 18.8 | 1 | 3.4 | 15 | 18.8 | 2 | 8.3 |  |
| Progressive disease/ relapse | 11 | 68.8 | 16 | 55.2 | 43 | 53.8 | 13 | 54.2 |  |
| Transplant-related mortality^β^ | 0 | 0 | 0 | 0 | 1 | 1.3 | 0 | 0 |  |

^α^ Early death defined as death within first 28 days ^β^ Transplant-related mortality defined as an death occuring after receiving allogeneic transplant. Definitions of causes of death were adapted from Slats et al.'s study (1).

# Treatment-related mortality predictors

To assess predictors of treatment-related mortality, we used 60-day mortality as a surrogate. Logistic regression was used to model predictors:

eTable 2 Multivariable logistic regression for 60-day mortality for AML patients treated with HMA with or without Venetoclax.

|  | Odds ratio | Lower CI | Upper CI | P-value |
| --- | --- | --- | --- | --- |
| (Intercept) | 0.045 | 0.001 | 2.017 | 0.110 |
| Azacitdine plus Venetolcax^α^ | 0.362 | 0.076 | 1.731 | 0.203 |
| Decitabine^α^ | 0.782 | 0.271 | 2.262 | 0.650 |
| Decitabine plus Venetolcax^α^ | 1.527 | 0.446 | 5.231 | 0.500 |
| Age_diagnosis | 1.025 | 0.987 | 1.064 | 0.200 |
| cytogenetic_category_ELN2017: Intermediate Risk^β^ | 0.575 | 0.041 | 8.095 | 0.682 |
| cytogenetic_category_ELN2017: Not performed/ Poor banding, Inadequate^β^ | 0.507 | 0.019 | 13.820 | 0.687 |
| cytogenetic_category_ELN2017: Unfavorable Risk | 0.318 | 0.017 | 5.873 | 0.441 |
| P53 mutated^γ^ | 4.542 | 1.442 | 14.302 | 0.010 |
| P53 wild type^γ^ | 2.386 | 0.906 | 6.286 | 0.078 |
| ECOG III-IV | 4.529 | 1.695 | 12.100 | 0.003 |

^α^ Compared to single-agent azacitidine. ^β^ Compared to favorable cytogenetics. ^γ^ Compared to P53 untested status.

# HMA vs. HMA with Venetoclax

*eFigure 1.* *Covariate balance before and after propensity score weighting for HMA vs. HMA-VEN subgroups*


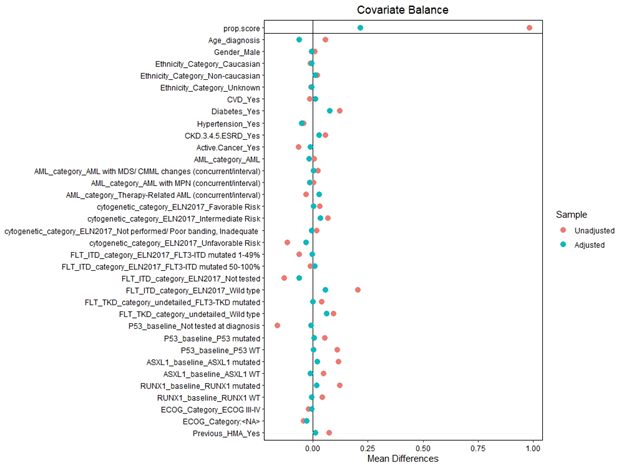


eTable 3. Adjusted Overall Survival at months 12 and 24 for AML patients treated with HMA vs. HMA plus Venetoclax

|  | strata | Time (months) | Survival | Lower CI | Upper CI |
| --- | --- | --- | --- | --- | --- |
| 1 | HMA | 12 | 0.36 | 0.28 | 0.46 |
| 2 | HMA | 24 | 0.22 | 0.15 | 0.31 |
| 3 | HMA plus Venetolcax | 12 | 0.40 | 0.27 | 0.60 |
| 4 | HMA plus Venetolcax | 24 | 0.21 | 0.11 | 0.41 |

eTable 4. Adjusted Event-free Survival at months 12 and 24 for AML patients treated with HMA vs. HMA plus Venetoclax

|  | strata | Time (months) | Survival | Lower CI | Upper CI |
| --- | --- | --- | --- | --- | --- |
| 1 | HMA | 12 | 0.23 | 0.16 | 0.32 |
| 2 | HMA | 24 | 0.13 | 0.08 | 0.22 |
| 3 | HMA plus Venetolcax | 12 | 0.28 | 0.17 | 0.48 |
|  | HMA plus Venetolcax | 24 | 0.22 | 0.12 | 0.42 |

Azacitidine vs. Azacitidine with Venetoclax

*eFigure 2. Covariate balance before and after propensity score weighting*


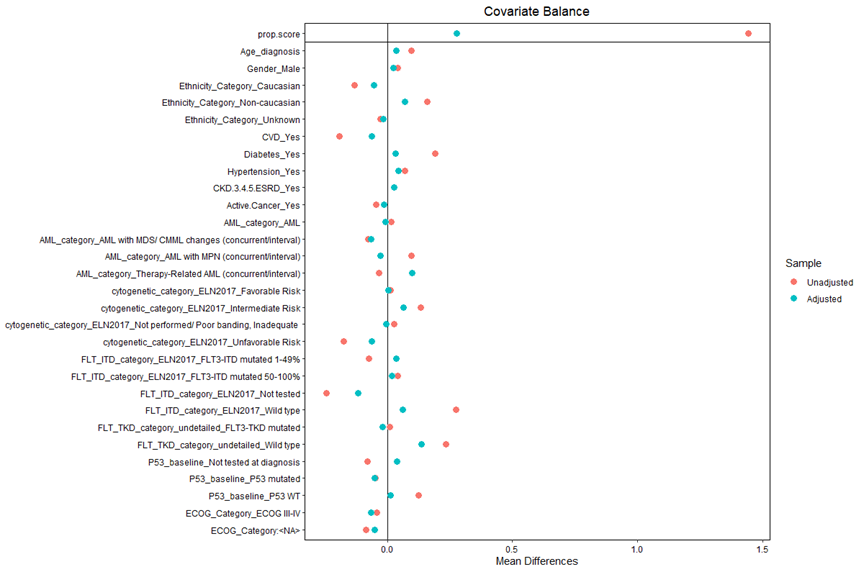


eTable 5 Adjusted Event-free Survival at months 12 and 24 for AML patients treated with Azacitidine vs. Azacitidine plus Venetoclax

|  | strata | Time (months) | Survival | Lower CI | Upper CI |
| --- | --- | --- | --- | --- | --- |
| 1 | Azacitdine plus Venetolcax | 12 | 0.40 | 0.21 | 0.77 |
| 2 | Azacitdine plus Venetolcax | 24 | 0.40 | 0.21 | 0.77 |
| 3 | Azactidine | 12 | 0.18 | 0.08 | 0.43 |
| 4 | Azactidine | 24 | 0.10 | 0.03 | 0.30 |

eFigure 3: Propensity score-adjusted Event-free Survival for AML patients treated with Azacitidine vs. Azacitidine plus Venetoclax


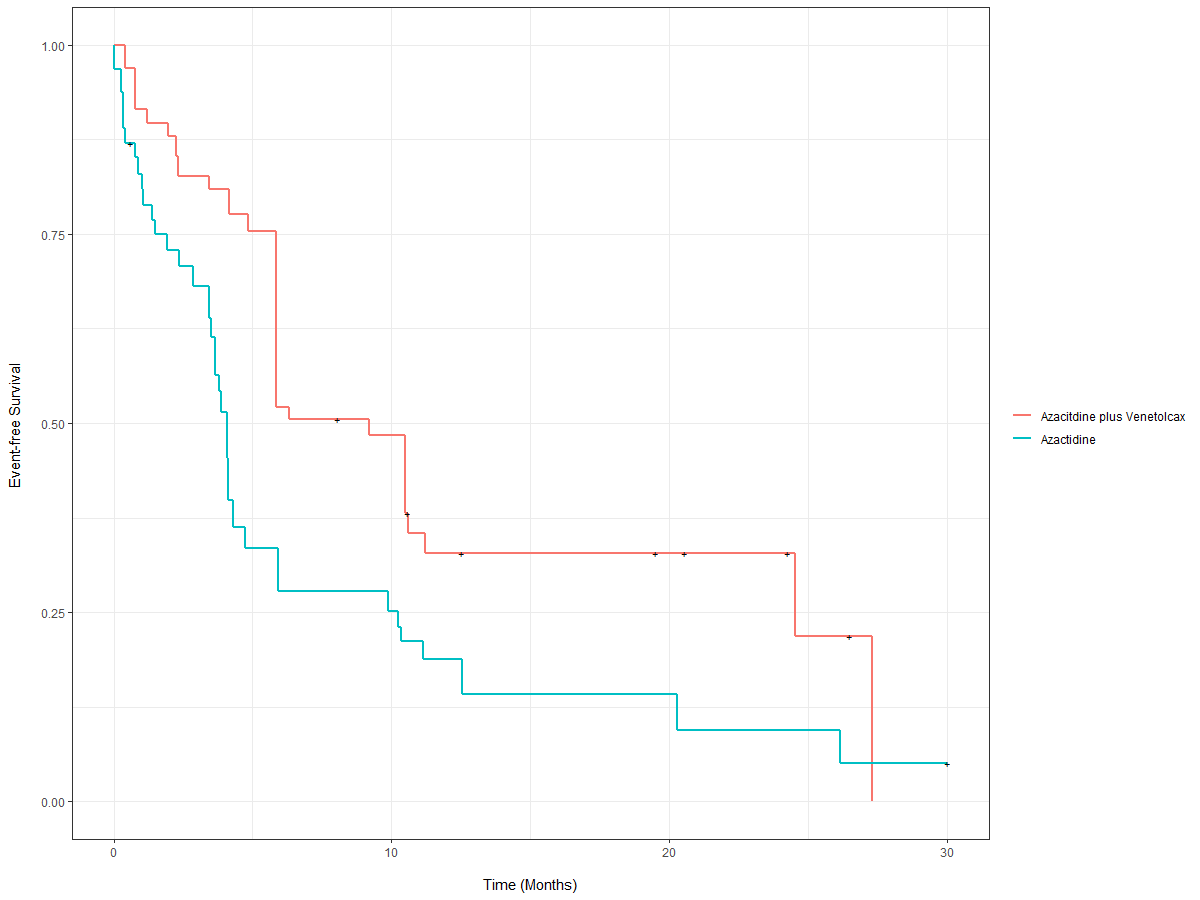


eTable 6. Adjusted Overall Survival at months 12 and 24 for AML patients treated with Azacitidine vs. Azacitidine plus Venetoclax

|  | strata | Time (months) | Survival | Lower CI | Upper CI |
| --- | --- | --- | --- | --- | --- |
| 1 | Azacitdine plus Venetolcax | 12 | 0.65 | 0.46 | 0.91 |
| 2 | Azacitdine plus Venetolcax | 24 | 0.39 | 0.20 | 0.78 |
| 3 | Azactidine | 12 | 0.41 | 0.26 | 0.64 |
| 4 | Azactidine | 24 | 0.28 | 0.15 | 0.53 |

eFigure 4: Propensity score-adjusted Overall Survival for AML patients treated with Azacitidine vs. Azacitidine plus Venetoclax


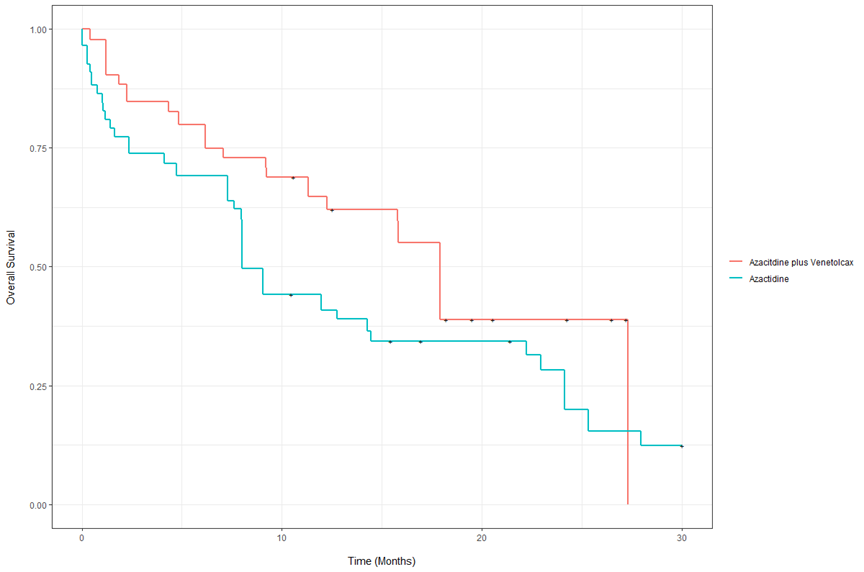


# Decitabine vs. Decitabine with Venetoclax

*eTable 7. Decitabine doses and schedules*

| Dose | Number | Proportion |
| --- | --- | --- |
| Decitabine 20 mg/m^2^ for five days | 7 | 0.08 |
| Decitabine 10 mg/m^2^ for ten days | 21 | 0.25 |
| Decitabine 10 mg/m^2^ for five days | 1 | 0.01 |
| Decitabine 20 mg/m^2^ for ten days | 55 | 0.65 |
|  |  |  |
| Ven + Decitabine 20 mg/m^2^ for five days | 3 | 0.11 |
| Ven + Decitabine 20 mg/m^2^ for ten days | 24 | 0.89 |

eFigure 5. Covariate balance before and after propensity score weighting in DEC vs. DEC-VEN patients


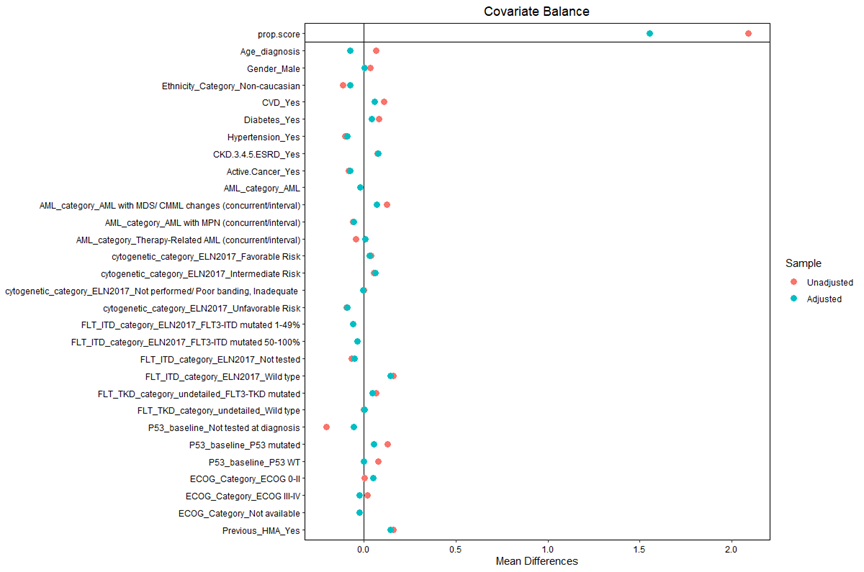


eTable 8 Adjusted Overall Survival at months 12 for AML patients treated with Decitabine vs. Decitabine plus Venetoclax

|  | strata | Time (months) | Survival | Lower CI | Upper CI |
| --- | --- | --- | --- | --- | --- |
| 1 | Decitabine | 12 | 0.34 | 0.17 | 0.68 |
| 3 | Decitabine plus Venetolcax | 12 | 0.26 | 0.13 | 0.51 |

eFigure 6. Propensity score-adjusted Overall Survival for AML patients treated with Decitabine vs. Decitabine plus Venetoclax


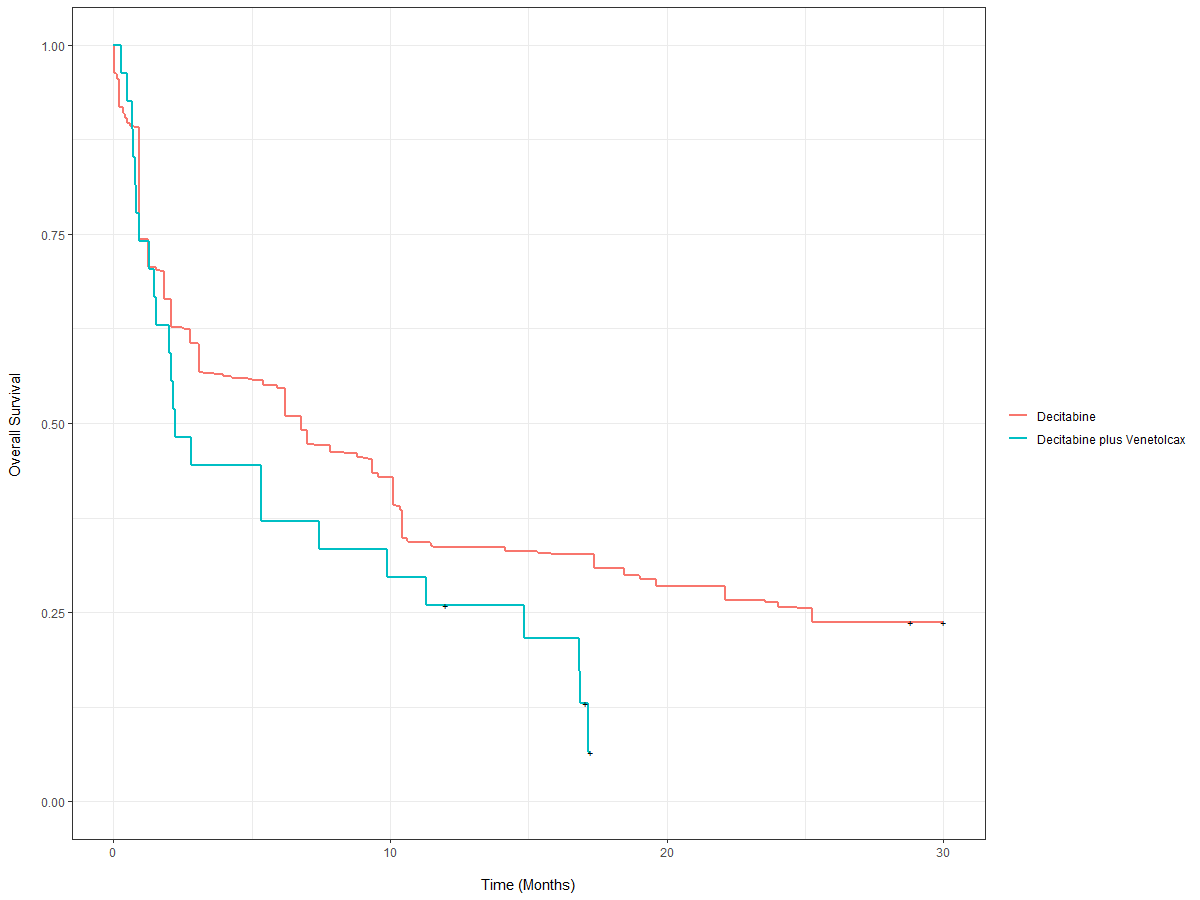


*eTable 9* *Adjusted Event-free Survival at months 12 for AML patients treated with Decitabine vs. Decitabine plus Venetoclax*

|  | strata | Time (months) | Survival | Lower CI | Upper CI |
| --- | --- | --- | --- | --- | --- |
| 1 | Decitabine | 12 | 0.32 | 0.24 | 0.44 |
| 3 | Decitabine plus Venetolcax | 12 | 0.21 | 0.10 | 0.43 |

eFigure 7 Propensity score-adjusted Event-free Survival for AML patients treated with Decitabine vs. Decitabine plus Venetoclax


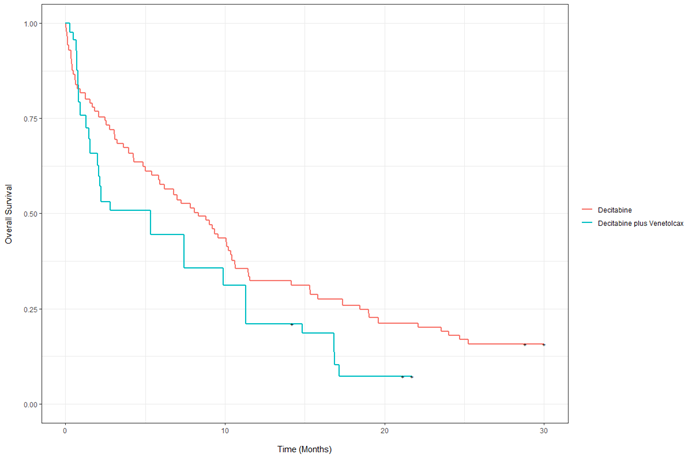


# Azacitidine vs. Decitabine

eFigure 8 Covariate balance before and after propensity score weighting


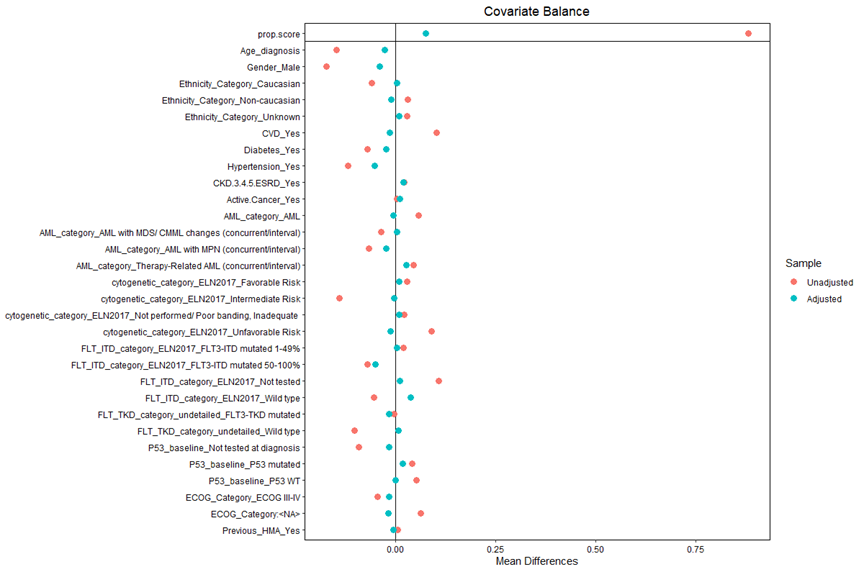


*eTable 10 Adjusted Overall Survival at months 12, 24, 36 and 48 for AML patients treated with Azacitidine vs. Decitabine*

|  | strata | Time (months) | Survival | Lower CI | Upper CI |
| --- | --- | --- | --- | --- | --- |
| 1 | Azactidine | 12 | 0.42 | 0.27 | 0.65 |
| 2 | Azactidine | 24 | 0.28 | 0.15 | 0.55 |
| 3 | Azactidine | 36 | 0.15 | 0.05 | 0.46 |
| 4 | Azactidine | 48 | 0.11 | 0.03 | 0.46 |
| 5 | Decitabine | 12 | 0.32 | 0.23 | 0.44 |
| 6 | Decitabine | 24 | 0.18 | 0.11 | 0.28 |
| 7 | Decitabine | 36 | 0.13 | 0.07 | 0.22 |
| 8 | Decitabine | 48 | 0.08 | 0.04 | 0.17 |

eFigure 9 Propensity score-adjusted Overall Survival for AML patients treated with Azacitidine vs. Decitabine


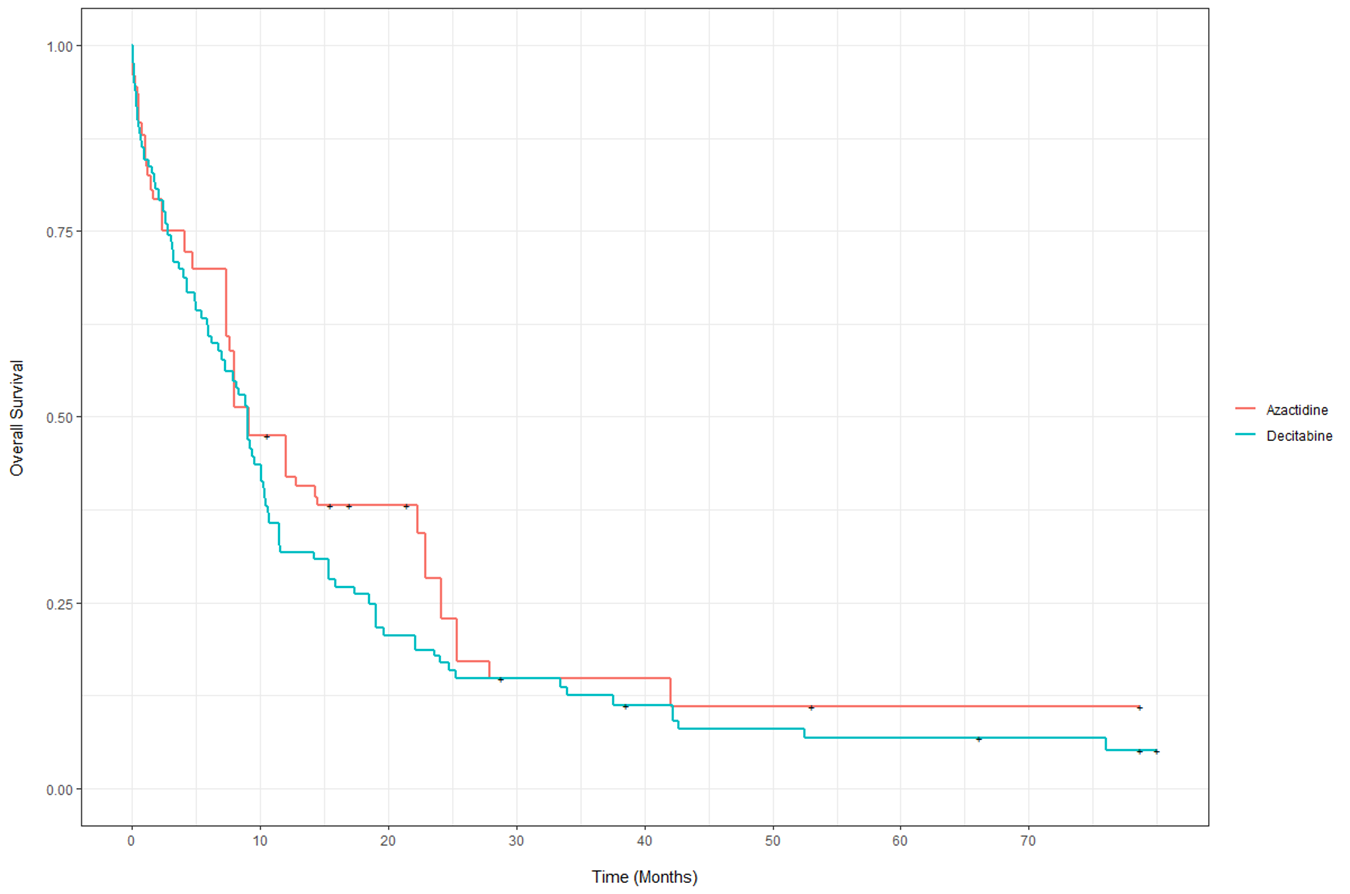


eTable 11 Adjusted Event-free Survival at months 12, 24, 36 and 48 for AML patients treated with Azacitidine vs. Decitabine

|  | strata | Time (months) | Survival | Lower CI | Upper CI |
| --- | --- | --- | --- | --- | --- |
| 1 | Azactidine | 12 | 0.192 | 0.083 | 0.445 |
| 2 | Azactidine | 24 | 0.103 | 0.030 | 0.348 |
| 3 | Azactidine | 36 | 0.016 | 0.002 | 0.113 |
| 4 | Azactidine | 48 | 0.016 | 0.002 | 0.113 |
| 5 | Decitabine | 12 | 0.259 | 0.176 | 0.381 |
| 6 | Decitabine | 24 | 0.144 | 0.083 | 0.250 |
| 7 | Decitabine | 36 | 0.090 | 0.042 | 0.191 |
| 8 | Decitabine | 48 | 0.069 | 0.030 | 0.160 |

eFigure 10 Propensity score-adjusted Event-free Survival for AML patients treated with Azacitidine vs. Decitabine


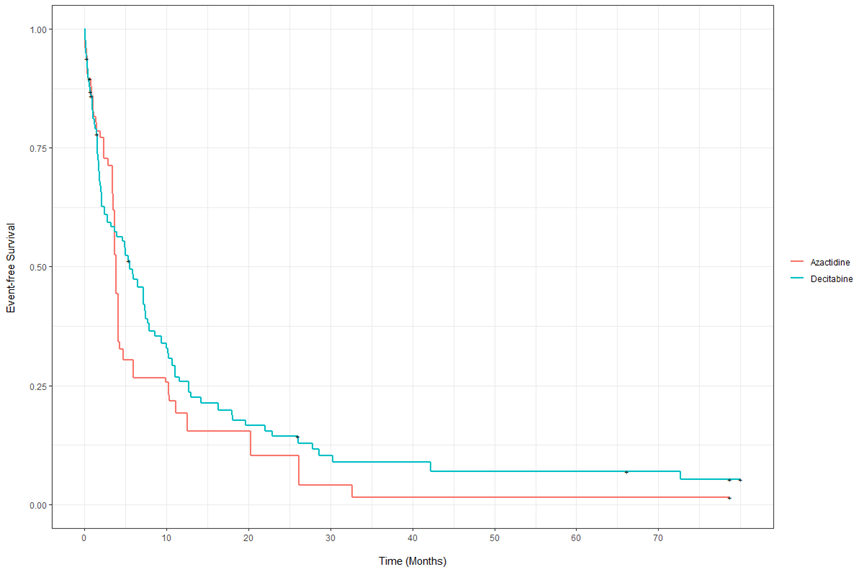


# Azacitidine plus Venbtoclax vs. Decitabine plus Venetoclax

eFigure 11 Covariate balance before and after propensity score weighting in AZA-VEN vs DEC-VEN


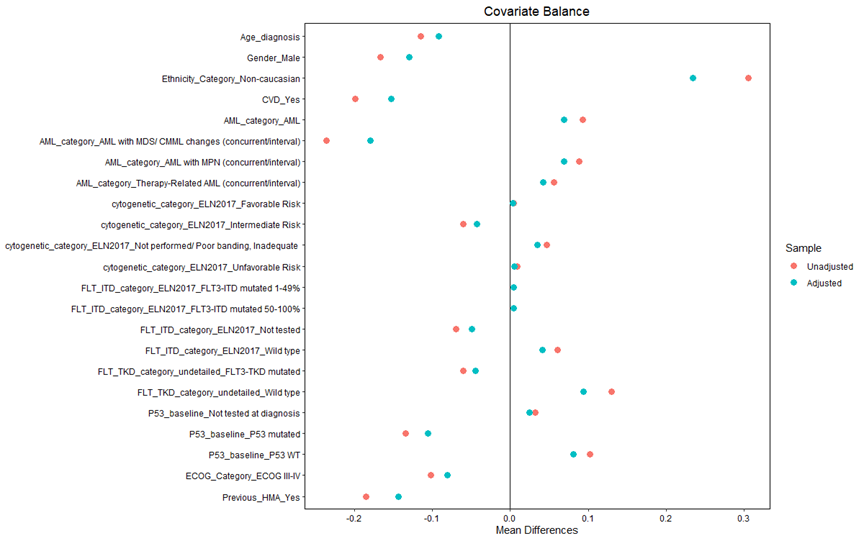


eTable 12 Adjusted Overall Survival at months 12 for AML patients treated with Azacitidine plus Venetoclax vs. Decitabine plus Venetoclax

|  | strata | Time (months) | Survival | Lower CI | Upper CI |
| --- | --- | --- | --- | --- | --- |
| 1 | Azacitdine plus Venetolcax | 12 | 0.54 | 0.37 | 0.78 |
| 2 | Decitabine plus Venetolcax | 12 | 0.28 | 0.13 | 0.62 |

eFigure 12: Propensity score-adjusted Overall Survival for AML patients treated with Azacitidine plus Venetoclax vs. Decitabine plus Venetoclax


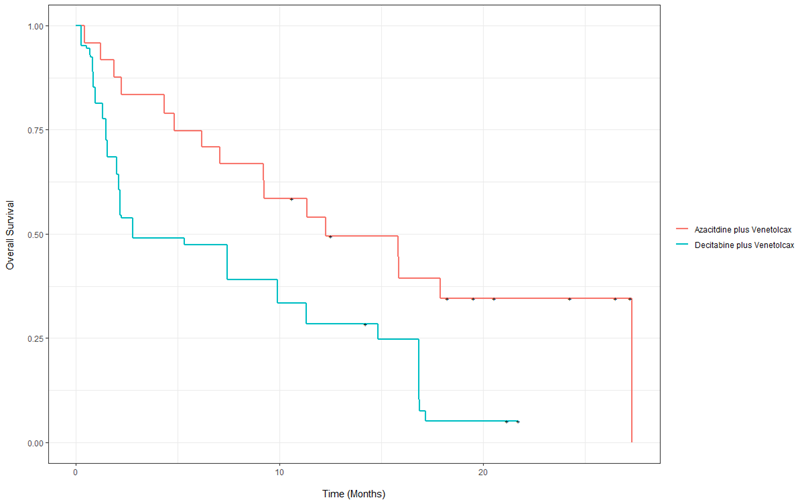


eTable 13 Adjusted Event-free Survival at months 12 for AML patients treated with Azacitidine plus Venetoclax vs. Decitabine plus Venetoclax

|  | strata | Time (months) | Survival | Lower CI | Upper CI |
| --- | --- | --- | --- | --- | --- |
| 1 | Azacitdine plus Venetolcax | 12 | 0.35 | 0.20 | 0.62 |
| 2 | Decitabine plus Venetolcax | 12 | 0.26 | 0.11 | 0.62 |

eFigure 13: Propensity score-adjusted Event-free Survival for AML patients treated with Azacitidine plus Venetoclax vs. Decitabine plus Venetoclax


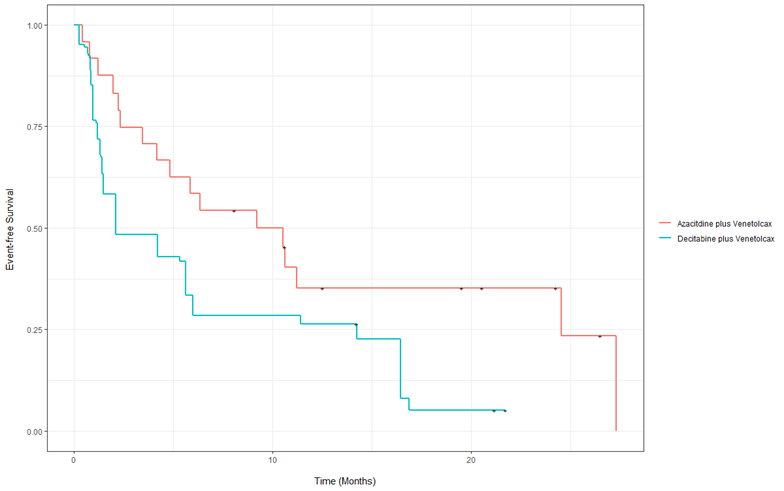


# Studies investigating the outcomes of combining Venetoclax with HMAs

*eTable 14 Studies investigating the role of venetoclax in combination with hypomethylating agents in newly diagnosed acute myeloid leukemia*

| Author/ Year | Study design/ setting | Study population | Comparison groups (number of patients) | Dose and schedule | Outcomes* | Remarks/ Comments |
| --- | --- | --- | --- | --- | --- | --- |
| DiNardo (1)/2019 | Phase Ib, open-label, uncontrolled Study/ Multi-institutional | All newly diagnosed AML patients unfit for induction chemotherapy because ≥ 65 years or coexisting conditions | AZA-VEN (73).  DEC-VEN (72). | AZA: 75 mg/m2 IV/SC for 7 days of 28 days cycle.  DEC: 20 mg/m2 IV for 5 days of 28 days cycle.  VEN: ramp up dose to 400, 800 or 1200 mg QD in escalation stage and 400 and 800 in expansion stage. | -HMA-VEN: m-OS: 17.5. CCR: 67%.  -AZA-VEN (400 mg): m-OS: NR. CCR: 76%.  -DEC-VEN (400 mg): m-OS: 14.2. CCR: 71%. | -Because of strict eligibility criteria and the uncontrolled nature of single-arm studies, outcomes have limited generalizability.  -Median follow-up was 8.9 mo. |
| Dinardo (2)/ 2020 | Phase III, Randomized, blinded, controlled Study/ Multi-institutional | All newly diagnosed AML patients unfit for induction chemotherapy because ≥ 75 years or coexisting conditions | AZA-Placebo (145) vs. AZA-VEN (286) | AZA: 75 mg/m2 IV/SC for 7 days of 28 days cycle. VEN: ramp up dose to 400 mg QD. | m-OS: 9.6 vs. 14.7, P<0.01. m-EFS: 7 vs. 9.8, P<0.01. CCR: 28.3% vs. 66.4%, P<0.001. | -Randomized trials control for both observable and unobservable confounding.  -Median follow-up was 20.5 mo. |
| Larbrador (3)/ 2020 | Retrospective cohort/ Database. Multi-institutional | Newly diagnosed AML patients unfit for induction | AZA (497) vs. DEC (141). | AZA: 75 mg/m2 IV/SC for 7 days of 28 days cycle.  DEC: 20 mg/m2 IV/SC for 5 days of 28 days cycle. | m-OS: 10 vs. 8, P=0.46. CCR: 18.5% vs. 22%, P=0.35. | -Outcomes were not adjusted. Median follow-up was 12 mo.  - Published in abstract form. |
| Morsia (4)/2020 | Retrospective cohort/ Single-center | All newly diagnosed and R/R AML patients treated with HMA with or without VEN. Patients ≥ 18 years. | - HMA vs. HMA-VEN (86).  - Newly diagnosed AML:  - HMA (56) vs. HMA-VEN (44). | AZA: 75 mg/m2 IV/SC for 7 days of 28 days cycle.  DEC: 20 mg/m2 IV/SC for 5 days of 28 days cycle. | In newly diagnosed AML: m-OS: 9.5 vs. 11, P=0.56. CCR: 23% vs. 50%, P<0.01. | -The HMA-VEN arm patients were matched with HMA patients. The method of matching was not specified. |
| Wen (5)/ 2020 | Network metanalysis | Newly diagnosed AML patients. Median age in groups was 73-75 | Direct HMA vs. conventional care regimen  Indirect comparison of AZA vs. DEC | AZA: 75 mg/m2 IV/SC for 7 days of 28 days cycle.  DEC: 20 mg/m2 IV/SC for 5 days of 28 days cycle. | AZA vs. DEC: relative mortality 0.83, P<0.01. Relative CR: 1.66, 95% CI 1.17–2.35. | -Indirect comparison using network metanalysis.  -Conventional care regimen included: LDAC, best supportive care and intensive chemotherapy. |
| Zeidan (6)/2020 | Phase III, Randomized, open-label, controlled Study/ Multi-institutional | All newly diagnosed AML patients unfit for induction chemotherapy because ≥ 75 years or coexisting conditions | Guadeictabine vs. AZA (171) /DEC (167/ LDAC. | AZA: 75 mg/m2 IV/SC for 7 days of 28 days cycle.  DEC: 20 mg/m2 IV/SC for 5 days of 28 days cycle. | AZA vs. DEC: m-OS: 8.7 vs. 8.2. CCR: 22.2% vs. 25.1%. | -Subsetted analysis from a randomized trial.  - Published in abstract form. |
| Bouligny (7)/ 2021 | Retrospective cohort/ Single-center | All newly diagnosed and relapsed AML patients treated with HMA or LDAC in combination with VEN. | Single-arm: AZA/DEC/LDAC-VEN (74) | Not specified | Newly diagnosed: CCR: 39%. Intermediate-risk AML m-OS: 13.7.  Adverse-risk AML m-OS: 9.2. | -No comparison was provided.  - Published in abstract form. |
| Garcia(8)/ 2021 | Retrospective cohort/ Multi-center | All newly diagnosed AML patients treated with VEN based regimen to non-VEN based regimens | VEN arm (113): AZA-VEN (80%), DEC-VEN (18%), LDAC-VEN (2%).  Non-VEN arm (113): AZA (14%), DEC (19.5%), Anthracycline-based (67%) | Not specified | 1-year-OS: 62.6% vs. 49.8% . 1-year-EFS: 41.4% vs. 31.5% .CCR: 60.4% vs. 50%. | -The non-VEN arm patients were matched with VEN patients. The method of matching was not specified. -Mean follow-up for VEN arm and non-VEN arm was 9.2 and 14.6 mo.  - Published in abstract form. |
| Kim (9)/2021 | Retrospective cohort/ Single-center | All newly diagnosed AML patients treated with DEC-VEN | -P53 mutated AML vs. P53 wild-type AML. Both groups were treated with DEC-VEN  - P53 mutated AML treated with VEN-DEC (35) vs. DEC (10). | DEC: 20 mg/m2 IV for 10 days of 28 days cycle until CR followed by 5-day cycles.  VEN: ramp up dose to 400 mg QD. | DEC vs, DEC-VEN: m-OS: 4.9 vs. 5.2. CCR: 53% vs. 66%. | -Comparisons groups DEC-VEN vs DEC are all P53 mutated.  -Outcomes were not adjusted. |
| Maiti (10)/ 2021 | Phase II, open-label, uncontrolled Study/ Single-institutional | Older patients with AML or high-risk myelodysplastic syndrome / chronic myelomonocytic leukemia | - Single-arm: DEC-VEN (219).  - Newly diagnosed AML (83).  - Untreated secondary AML (20).  - Treated secondary AML (25). | DEC: 20 mg/m2 IV for 10 days of 28 days cycle until CR followed by 5-day cycles.  VEN: ramp up dose to 400 mg QD. | - Newly diagnosed AML: m-OS: 16.2, CCR: 83% .  - Untreated secondary AML: m-OS: 10.7, CCR: 65%.  - Treated secondary AML : m-OS: 5.8, CCR: 40%. | -No comparison was provided. No statistical test was used for comparison between groups.  -Median follow-up was 24.7 mo.  - Published in abstract form. |
| Patel (11)/ 2021 | Retrospective cohort/ Database | All newly diagnosed AML patients treated with HMA with VEN. | AZA-VEN (54).  DEC-VEN (52).  LDAC-VEN (6). | Not specified | AZA-VEN vs. DEC-VEN vs. LDAC-VEN: m-OS: 11.3 vs. 13.9 vs. 6.5, P=0.77. ORR: 55.6%, 57.7%, 33.3%, P=0.85. | -Outcomes were not adjusted.  - Published in abstract form. |
| Current study/ 2021 | Retrospective cohort/ Single-center | All newly diagnosed AML patients treated with HMA with or without VEN. Patients ≥ 18 years. | - HMA (119) vs. HMA-VEN (51)  -AZA (35) vs. AZA-VEN (24)  -DEC (84) vs. DEC-VEN (27)  -AZA (35) vs. DEC (84)  -AZA-VEN (24) vs. DEC-VEN: | AZA: 75 mg/m2 IV/SC for 7 days of 28 days cycle.  DEC: 10-20 mg/m2 IV for 5-10 days of 28 days cycle.  VEN: ramp up dose to 400 mg QD. | -HMA vs. HMA-VEN^α^: m-OS: 8.8 vs. 7.43, P=0.7. m-EFS: 4.13 vs. 4.2, P=0.6. CCR: 27% vs. 52%, P< 0.01.  -Aza vs. AZA-VEN^α^: m-OS: 8 vs. 17.9, P=0.26. m-EFS: 3.8 vs. 10.5, P=0.02. CCR: 10% vs. 54%, P< 0.01.  -DEC vs. DEC-VEN^α^: m-OS: 8.3 vs. 5.3, P=0.07. m-EFS: 5 vs. 2.1, P=0.16. CCR: 32% vs. 43%, P=0.35.  -AZA vs. DEC^α^: m-OS: 9 vs. 9, P=0.3. m-EFS: 3.8 vs. 5.5, P= 0.6. CCR: 13% vs. 33%, P=0.04.  -AZA-VEN vs. DEC-VEN^α^: m-OS: 12.3 vs. 2.8, P=0.02. m-EFS: 9.2 vs. 2.1 , P=0.02. CCR: 58% vs. 52%, P=0.66. | -We used propensity score to adjust for observable confounding. Average treatment effect obtained.  -In DEC only arm, 65% received 20 mg/m2 IV for 10 days of 28 days cycle until CR followed by 5 day cycles and 25% received 10 mg/m2 for 10 days. In DEC-AZA arm, 89% received 20 mg/m2 IV for 10 days of 28 days cycle until CR followed by 5-day cycles.  -Median follow-up for HMA and HMA-VEN was 78.6 and 21.13 mo. |

AML: acute myeloid leukemia; AZA: azacitidine; VEN: venetoclax; DEC: decitabine; IV: intravenous; SC: subcutaneous, QD: daily; CCR: composite complete response; m-OS: median overall survival; mo: months; LDAC: low dose cytarabine; NR: not reached; ORR: overall response rate; m-EFS: median event-free survival; R/R: relapsed refractory. *Follow-up expressed in months. ^α^ Outcomes adjusted using propensity score weighting.

# References:

1. DiNardo CD, Pratz K, Pullarkat V, Jonas BA, Arellano M, Becker PS, et al. Venetoclax combined with decitabine or azacitidine in treatment-naive, elderly patients with acute myeloid leukemia. Blood. 2019;133(1):7-17.

2. DiNardo CD, Jonas BA, Pullarkat V, Thirman MJ, Garcia JS, Wei AH, et al. Azacitidine and Venetoclax in Previously Untreated Acute Myeloid Leukemia. New England Journal of Medicine. 2020;383(7):617-29.

3. Labrador J, Martínez-Cuadrón D, de la Fuente A, Rodriguez-Veiga R, Serrano J, Tormo M, et al. Azacitidine Vs. Decitabine in Unfit Newly Diagnosed Acute Myeloid Leukemia Patients: Results from the Pethema Registry. Blood. 2020;136:25-7.

4. Morsia E, McCullough K, Joshi M, Cook J, Alkhateeb HB, Al-Kali A, et al. Venetoclax and hypomethylating agents in acute myeloid leukemia: Mayo Clinic series on 86 patients. American Journal of Hematology. 2020;95(12):1511-21.

5. Wen B, You W, Yang S, Du X. Indirect comparison of azacitidine and decitabine for the therapy of elderly patients with acute myeloid leukemia: a systematic review and network meta-analysis. Experimental hematology & oncology. 2020;9:3.

6. Zeidan A, Fenaux P, Gobbi M. Comparative results of azacitidine and decitabine from a large prospective phase 3 study in treatment naive patients with acute myeloid leukemia not eligible for intensive chemotherapy [ASH abstract 1037]. Blood. 2020;136(1).

7. Bouligny I, Maher K. Outcomes of Induction with Venetoclax in Combination with Decitabine, Azacitidine, or Low-Dose Cytarabine for Treatment of AML: A Real-World Retrospective Analysis [ASH abstract 2335]. 2021.

8. Garcia JS, Wolach O, Vachhani P, Zeidner JF, Talati C, Pollyea DA, et al. Comparative Effectiveness of Venetoclax Combinations Vs Other Therapies Among Patients with Newly Diagnosed Acute Myeloid Leukemia: Results from the AML Real World Evidence (ARC) Initiative [ASH abstract 2328]. 2021.

9. Kim K, Maiti A, Loghavi S, Pourebrahim R, Kadia TM, Rausch CR, et al. Outcomes of TP53-mutant acute myeloid leukemia with decitabine and venetoclax. Cancer. 2021;127(20):3772-81.

10. Maiti A, DiNardo C, Rausch C, Pemmaraju N, Garcia-Manero G, Ohanian M, et al. Ten-Day Decitabine with Venetoclax (DEC10-VEN) in Acute Myeloid Leukemia and Myelodysplastic Syndrome: Updated Results of a Phase II Trial [ASH abstract 1270]. 2021.

11. Patel P, Madanat Y, Belli A, Hansen E, Foss H, Schulte M, et al. Frontline Venetoclax (ven) Based Combination Therapy in Older Adults with Acute Myeloid Leukemia Treated in the Real-World Setting; A Multi-Institutional Retrospective Study [ASH abstract 4136]. 2021.
